# New syntheses of (±)-tashiromine and (±)-epitashiromine via enaminone intermediates

**DOI:** 10.3762/bjoc.12.256

**Published:** 2016-12-02

**Authors:** Darren L Riley, Joseph P Michael, Charles B de Koning

**Affiliations:** 1 Department of Chemistry, University of Pretoria, Pretoria 0028, South Africa; 2 Molecular Sciences Institute, School of Chemistry, University of the Witwatersrand, Wits 2050, Johannesburg, South Africa

**Keywords:** alkaloid, enaminone, epitashiromine, indolizidine, tashiromine

## Abstract

The syntheses of the naturally occurring indolizidine alkaloid (±)-tashiromine and its unnatural epimer (±)-epitashiromine are demonstrated through the use of enaminone chemistry. The impact of various electron-withdrawing substituents at the C-8 position of the indolizidine core on the preparation of the bicyclic system is described.

## Introduction

**Figure 1:**
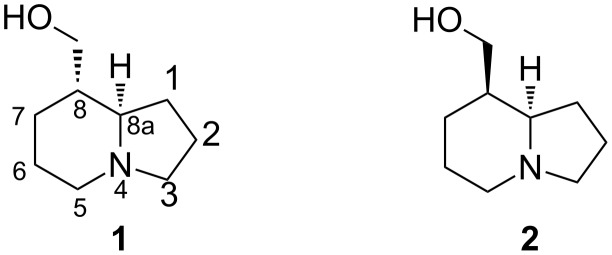
Structures of (±)-tashiromine (**1**) and (±)-epitashiromine (**2**) showing the systematic numbering of the indolizidine skeleton. The epimer **2** is often referred to as 5-epitashiromine, in accordance with the alternative numbering of the bicyclic system as a 1-azabicyclo[4.3.0]nonane.

Tashiromine (**1**) is a monosubstituted indolizidine alkaloid originally isolated from the Asian deciduous shrub *Maackia tashiroi* [1] and subsequently from the genera *Poecilanthe* [2] and *Crotalaria* [3] (Figure 1). To date this natural product and its unnatural epimer **2** have been the targets of numerous enantioselective and racemic syntheses [4–9]. Typical approaches to accessing the alkaloid’s indolizidine skeleton have included a key cyclisation onto the nitrogen atom of either a piperidine or a pyrrolidine-based precursor, with few reports of alternative approaches.

The approach adopted by our research group to the synthesis of these and related bicyclic alkaloids takes advantage of enaminone chemistry [10–19]. Our interest in the enaminone manifold stems mainly from its ability to display both ambident nucleophilic and electrophilic reactivity, properties that have frequently been exploited in the synthesis of alkaloids and other nitrogen-containing heterocycles [20–23]. The enaminone and related units that form the basis of our own approach most commonly comprise a nitrogen atom in a pyrrolidine or piperidine ring conjugated at C-2 through an exocyclic vinyl fragment to an ester (vinylogous urethane) or another electron-withdrawing substituent (usually ketone, nitrile, nitro or sulfone). When suitable carbon chains bearing terminal electrophilic substituents are attached to the nitrogen, alkylating or acylating cyclisation onto the enamine carbon site leads to the formation of bicyclic systems with nitrogen located at the bridgehead. Our approach to the synthesis of indolizidines is thus unusual in that it entails the formation of the C-7/C-8 bond. In this article we describe further extensions to our route to substituted indolizidines, and an application to the synthesis of racemic tashiromine (**1**) and epitashiromine (**2**).

## Results and Discussion

**Scheme 1:**
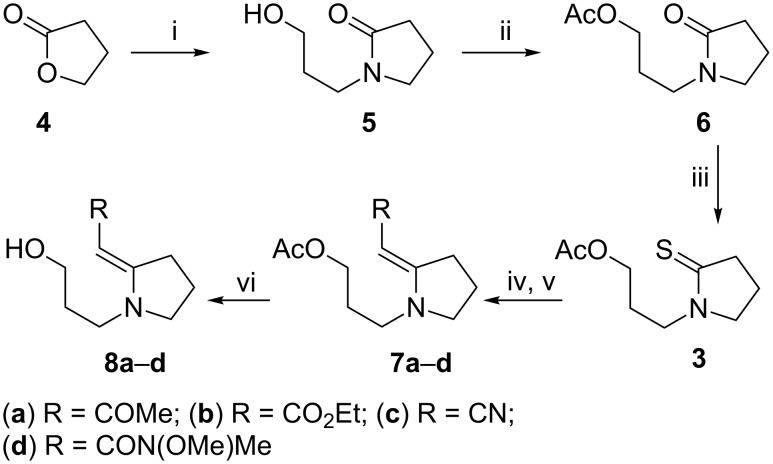
Reagents and conditions: (i) NH_2_(CH_2_)_3_OH, 250 °C (sealed tube), 18 h, 81%; (ii) Ac_2_O, pyridine, 0 °C, 10 min, then, rt, 18 h, 87%; (iii) Na_2_CO_3_, P_2_S_5_, THF, 5 h, 90%; (iv) BrCH_2_R, CH_3_CN, rt, 24 h; (v) PPh_3_, NEt_3_, CH_3_CN, 5 h; (vi) K_2_CO_3_, MeOH, rt, 3 h.

One of the most versatile methods for preparing the enaminones of interest is the Eschenmoser sulfide contraction of thiolactams with α-halocarbonyl compounds [24–25], which was originally described by Eschenmoser and co-workers in 1970–1971 [26–27]. We were interested to examine the impact of different electron-withdrawing substituents at what would become the 8-position of the indolizidine skeleton. To this end we chose to investigate the reaction of 3-(2-thioxo-1-pyrrolidinyl)propyl acetate (**3**), which we had previously used as a precursor to enaminones and indolizidines [28], with three different reaction partners, namely, α-bromoacetone, ethyl α-bromoacetate and α-bromoacetonitrile. The preparation of **3** in three steps from commercially available 3-amino-1-propanol and γ-butyrolactone (**4**) and its subsequent transformation into the substituted enamines is shown in Scheme 1.

As described previously [28], 3-amino-1-propanol was condensed with γ-butyrolactone (**4**) in a sealed Carius tube at 250 °C, affording alcohol **5** in 81% yield. The subsequent protection of the primary alcohol as acetate **6** (87% yield), followed by thionation with phosphorus pentasulfide using the Brillon procedure [29] afforded thiolactam **3** in 90% yield. The succeeding Eschenmoser reaction with α-halocarbonyl compounds afforded enaminones **7a** and **7b** in good yields (90–95%), but the reaction with α-bromoacetonitrile gave enaminonitrile **7c** in only 44% yield. The deacetylation of **7a**–**c** with potassium carbonate in methanol afforded the corresponding alcohols **8a**–**c** in good yields (82–89%; Table 1). The data for the vinylogous urethane **8b** agreed with those for the same compound which we had previously prepared by alternative methods [13–14]. For comparison, Table 1 also includes our previously reported results [19] with an α-bromo Weinreb acetamide, 2-bromo-*N*-methoxy-*N*-methylacetamide.

**Table 1:** Yields for compounds shown in Scheme 1 and Scheme 2.

R = COMe (yield, %)	R = CO_2_Et (yield, %)	R = CN (yield, %)	R = CO[N(OMe)Me] (yield, %) [19]

**7a** (95)	**7b** (90)	**7c** (44)	**7d** (85)
**8a** (82)	**8b** (85)	**8c** (89)	**8d** (83)
**9a** (27)^a^	**9b** (59)	**9c** (72)	**9d** (64)
**12a** (0)	**12b** (72; dr 85:15)	**12c** (85; dr 92:8)^b^	**12d** (25; dr 95:5)^c^

^a^Contaminated with triphenylphosphine traces. ^b^Best result; reduction not reproducible. ^c^Based on the *N*-hydroxypropyl enaminone **8d**.

**Scheme 2:**
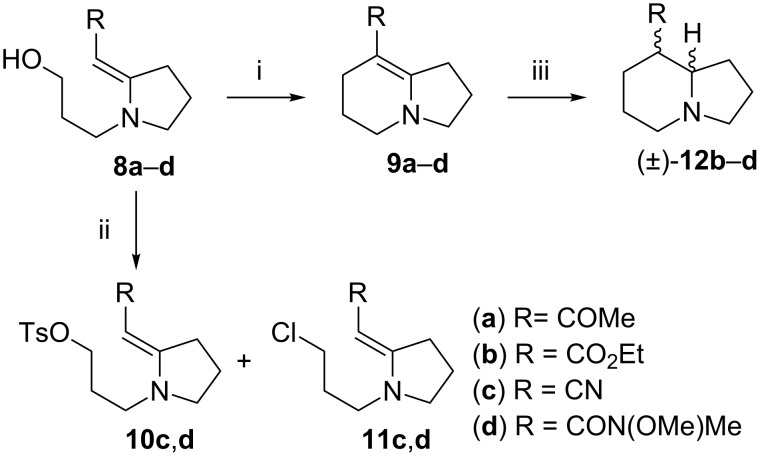
Reagents and conditions: (i) Imidazole, PPh_3_, I_2_, CH_3_CN–PhCH_3_, reflux, 1 h; (ii) *p*-TsCl, NEt_3_, DMAP, CH_2_Cl_2_, rt, 18 h, (**10c**) 19%, (**10d**) 71%; (iii) H_2_ (1 atm), Adams catalyst, AcOH, rt, 24 h.

A key step in the planned reaction sequence was the alkylating cyclisation of the liberated alcohols **8a**–**c** to produce the indolizidine nucleus (Scheme 2). The cyclisation was achieved by initially treating these deprotected enaminones with imidazole and triphenylphosphine at ambient temperature in acetonitrile followed by iodine under refluxing conditions [30]. The products from vinylogous urethane **8b** and vinylogous cyanamide **8c** were easily purified under standard chromatographic conditions, affording the corresponding bicyclic systems **9b** and **9c** in 59% and 72% yields, respectively (Scheme 2 and Table 1). We had previously found that the bicyclic vinylogous urea **9d** (64%) required a more careful flash chromatographic separation [19]. Unfortunately, the bicyclic vinylogous amide **9a** could not be separated adequately from the triphenylphosphine residues under standard chromatographic and recrystallisation conditions. In an attempt to circumvent the issues associated with the purification of the cyclic enamines, we investigated the conversion of alcohols **8c** and **8d** into the corresponding tosylates followed by treatment with sodium iodide as an alternative for the cyclisation step. The tosylations afforded **10c** and **10d** in 19% and 71% yields, respectively, with small amounts of the corresponding chlorides **11c** and **11d** present. However, we were unable to facilitate the desired cyclisation of these compounds to access the indolizidine skeleton, even after in situ conversion of the intermediates into the corresponding iodides. We reverted to using the original procedure, but adjusted the purification protocol by first separating both the products and triphenylphosphine oxide residues from the baseline impurities by column chromatography, then removing most of the remaining triphenylphosphine residues by simple recrystallisation from hexane.

The cyclised enaminones **9b**–**d** underwent catalytic hydrogenation in the presence of Adams catalyst (PtO_2_·xH_2_O) under mildly acidic conditions. The reduction can proceed either by direct *cis*-hydrogenation of the C=C bond, or by hydrogenation of the bicyclic iminium system formed by C-protonation of the enaminone. In the latter case, the preferred formation of the product with a *cis* relationship of the hydrogen atoms between C-8 and C-8a in the free base has been argued on stereoelectronic grounds [31], although the formation of some *trans*-product cannot be precluded. In the event, the products **12b**–**d** were obtained as racemic mixtures of diastereomers in which, as expected, the major diastereomer resulted from *cis* addition of hydrogen across the double bond. This was confirmed in the case of the reduced ester **12b**, which was obtained in a good yield of 72% and a diastereomeric ratio of 85:15. The NMR spectroscopic data for the two diastereomers (8R*,8aR*)-**12b'** and (8R*,8aS*)-**12b"**, pure samples of which could be obtained for characterisation by careful chromatography, agreed with those reported for the same isomers by Kiss et al. [9].

The reduction of the alkene unit in the bicyclic vinylogous cyanamide **9c** was not reproducible. In the best case we isolated the nitrile **12c** in 85% yield. Despite repeating the reaction several times and altering reaction times and hydrogen pressure in an attempt to optimise the reaction conditions, in most cases the nitrile group also appeared to be fully or partially reduced. In the case of the reduction of **9a**, which still had significant triphenylphosphine residues present, we were unable to isolate any product. Interestingly, we were also never able to isolate the reduced product **12d** when the bicyclic vinylogous urea **9d** contained anything more than trace amounts of triphenylphosphine residues; our best yield for the product was only 25% [19].

**Scheme 3:**
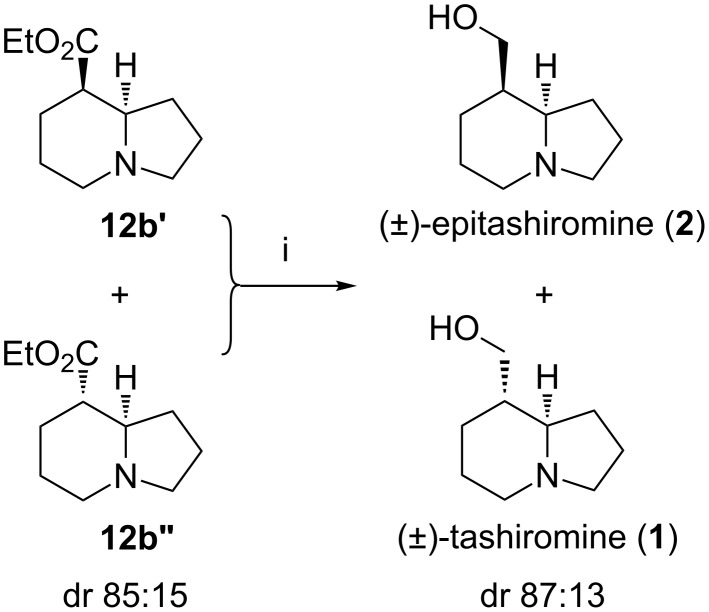
Reagents and conditions: (i) LiAlH_4_, Et_2_O, 3 h, 87%.

Reduction of the diastereomeric mixture of esters **12b** to the corresponding alcohols was achieved with a slurry of lithium aluminium hydride in diethyl ether. The reduction afforded a mixture of (±)-tashiromine (**1**) and its epimer (±)-epitashiromine (**2**) in an overall yield of 87% and in a 13:87 ratio, which matches the diastereoisomeric ratio in the precursor (Scheme 3). Pure samples for characterisation could be obtained by flash column chromatography on silica gel using a 95:4.75:0.25 mixture of methanol/dichloromethane/ammonium hydroxide. The spectroscopic data compared well with previously published results [7]. In particular, ^13^C chemical shifts for both tashiromine and epitashiromine were within ±1.0 ppm of those reported by Dieter et al. [32] and Kim et al. [33].

## Conclusion

A concise total synthesis of (±)-tashiromine (**1**) and (±)-epitashiromine (**2**) using enaminone chemistry is reported. The NMR spectroscopic data correlated well with those in previously reported syntheses. The synthetic approach indicated that there was reasonable tolerance of functionality at the 8-position, with only the nitrile experiencing reproducibility issues during the cyclisation step. Key to the success of the synthesis however is the ability to remove efficiently the triphenylphosphine residues after the cyclisation step as the catalytic hydrogenation appeared to be adversely effected by the presence of these residues.

## Supporting Information

File 1 Experimental procedures and copies of NMR spectra.
