# Supplementary material for: New syntheses of (±)-tashiromine and (±)-epitashiromine via enaminone intermediates
Source: Beilstein J Org Chem. 2016 Dec 2;12:2609–13. doi: 10.3762/bjoc.12.256 (PMC5238587; doi:10.3762/bjoc.12.256)

Supporting Information

for

New syntheses of (±)-tashiromine and (±)-epitashiromine via enaminone intermediates

Darren L. Riley[1,2,*], Joseph P. Michael[2,*] and Charles B. de Koning[2]

Address: [1]Department of Chemistry, University of Pretoria, Pretoria 0028, South Africa and [2]Molecular Sciences Institute, School of Chemistry, University of the Witwatersrand, Wits 2050, Johannesburg, South Africa

Email: Darren L. Riley - darren.riley@up.ac.za; Joseph P. Michael - joseph.michael@wits.ac.za

*Corresponding author

Experimental procedures and copies of NMR spectra

Experimental S2

References S16

^{1}H and ^{13}C NMR spectra:

3-[(2*E*)-2-(2-Oxopropylidene)pyrrolidinyl]propyl acetate (**7a**) S17

Ethyl (2*E*)-{1-[3-(acetoxy)propyl]-2-pyrrolidinylidene}ethanoate (**7b**) S18

3-[(2*E*)-2-(Cyanomethylene)pyrrolidinyl]propyl acetate (**7c**). S19

(1*E*)-1-[1-(3-Hydroxypropyl)-2-pyrrolidinylidene]-2-propanone (**8a**) S20

Ethyl (2*E*)-[1-(3-hydroxypropyl)-2-pyrrolidinylidene]ethanoate (**8b**) S21

(2*E*)-[1-(3-Hydroxypropyl)-2-pyrrolidinylidene]ethanenitrile (**8c**) S22

Ethyl 1,2,3,5,6,7-hexahydroindolizine-8-carboxylate (**9b**)...................................... S23
1,2,3,5,6,7-Hexahydroindolizine-8-carbonitrile (**9c**)... S24
3-[(2*E*)-2-(Cyanomethylene)pyrrolidinyl]propyl 4-methylbenzenesulfonate (**10c**)... S25
(2*E*)-[1-(3-Chloropropyl)-2-pyrrolidinylidene]ethanenitrile (**11c**)............................. S26
3-((2*E*)-2-{2-[Methoxy(methyl)amino]-2-oxoethylidene}pyrrolidinyl)propyl 4-methylbenzenesulfonate (**10d**) and (2*E*)-2-[1-(3-Chloro-propyl)-2-pyrrolidinylidene]-*N*-methoxy-*N*-methylethanamide (**11d**)................................ S27
Ethyl (8*R**,8a*R**)-octahydroindolizine-8-carboxylate (**12b'**) S28
Ethyl (8*R**,8a*S**)-octa-hydroindolizine-8-carboxylate (**12b"**)................................... S29
Octahydroindolizine-8-carbonitrile diastereomers (**12c**) S30
(±)-Tashiromine (**1**) and (±)-epitashiromine (**2**) (mixture).. S31
(±)-Tashiromine (**1**)... S32
(±)-Epitashiromine (**2**)... S33

Experimental

1.1 General

All reagents used for reactions and preparative chromatography were distilled. Solvents used in reactions were pre-dried in their reagent bottles and then distilled over the appropriate drying medium under a nitrogen atmosphere. Acetonitrile, dichloromethane and methanol were distilled from calcium hydride. Triethylamine was distilled from, and stored over, potassium hydroxide. Acetic anhydride was distilled before storage over 4 Å molecular sieves. *p*-Toluenesulfonyl chloride was purified according to Perrin et al. [1] before use, and stored in a desiccator until required. All reactions were performed under an inert atmosphere (either dry nitrogen or argon) using a standard manifold line connected to a vacuum pump. The R_f values quoted are for thin layer chromatography (TLC) on aluminium-backed Macherey-Nagel ALUGRAMSil G/UV254 plates pre-coated with 0.25 mm silica gel 60, or Aldrich TLC plates (silica gel on aluminium). Macherey-Nagel Silica gel 60 (particle

size 0.063–0.200 mm) was used as the adsorbent for conventional preparative column chromatography, with a silica to product ratio of 30:1. The elution process was performed using the indicated solvent mixtures either under gravity or air pump pressure conditions. Whatman Partisil Prep 40 (particle size 0.040–0.063 mm) was used for preparative flash chromatography. Concentration or evaporation in vacuo refers to the removal of solvent under reduced pressure (~20 mm Hg, 45 °C) on a rotary evaporator and final drying on an oil pump (~1–2 mm Hg) at room temperature. Intermediates **3**, **5** and **6** were prepared as described previously [2].

All melting points were obtained on a Reichert hot-stage microscope, and are uncorrected. Infrared spectra were obtained on a Bruker Vector 22 spectrometer, or a Varian 800FTIR spectrometer (Scimitar Series). The absorptions are reported on the wavenumber (cm^{-1}) scale, in the range 400–4000 cm^{-1}. Hydrogen (^{1}H NMR) and carbon (^{13}C NMR) nuclear magnetic resonance spectra were recorded on a Bruker Avance-300 instrument at 300.13 MHz and 75 MHz, respectively using standard pulse sequences. The probe temperature for all experiments was 300±1 K. All spectra were recorded in deuterated chloroform ($CDCl_3$) in 5 mm NMR tubes. Chemical shifts are reported in parts per million (ppm) relative to tetramethylsilane as internal standard in the case of ^{1}H NMR spectra, and relative to the central signal of deuterated chloroform taken at δ 77.16 for the ^{13}C NMR spectra. High-resolution mass spectra were recorded on a VG7-SEQ Double Focussing Mass Spectrometer at 70 eV and 200 mA. The polarity was positive, ionisation employed was EI with a resolution of 3000, a mass range of 3000 amu (8 kV) and a scan rate of 5 s/decade.

*1.2 General procedure for the sulfide contraction of 3-(2-thioxo-1-pyrrolidinyl)propyl acetate (**3**)*

The thiolactam **3** (1 equiv) [2] and the relevant halide (1.05 equiv) were stirred at rt in dry CH_2Cl_2 (2 mL mmol^{-1}) for 5 h. The solvent was removed under high vacuum, and the resulting salt was stirred at rt for 18 h to complete the reaction. The salt was dissolved in MeCN (3 mL mmol^{-1}), to which was added a solution of PPh_3 (1.05 equiv) and dry NEt_3 (1.05 equiv) in MeCN (3 mL mmol^{-1}). The mixture was then stirred at rt for 5 h, during which time a white precipitate was formed. The solution was filtered through a pad of celite and evaporated *in vacuo*. The residue was taken up in EtOAc (10 mL mmol^{-1}), triturated for 30 min and again filtered through a pad of celite. The filtrate was extracted with HCl (2 M, 3 × 10 mL mmol^{-1}), the aqueous extracts were brought to pH 11 with aq. NH_3 solution (35%) and back-extracted with CH_2Cl_2 (3 × 10 mL mmol^{-1}). The organic extracts were combined, dried ($MgSO_4$), filtered and evaporated *in vacuo* to yield the crude products **7**. The products were purified by column chromatography on silica gel.

*1.3 3-[(2E)-2-(2-Oxopropylidene)pyrrolidinyl]propyl acetate (**7a**)*

3-(2-Thioxo-1-pyrrolidinyl)propyl acetate (**3**, 1.03 g, 5.09 mmol) and bromoacetone (0.733 g, 0.45 mL, 5.35 mmol) were allowed to react in dry CH_2Cl_2 (10 mL) followed by treatment with PPh_3 (1.41 g, 5.35 mmol) and NEt_3 (0.541 g, 0.750 mL, 5.35 mmol) in MeCN (15.5 mL) according to the general procedure, after which time the standard work-up and purification yielded 3-[(2*E*)-2-(2-oxopropylidene)pyrrolidinyl]propyl acetate (**7a**) as a light yellow oil (1.09 g, 95%); R_f 0.28 (CH_3OH:CH_2Cl_2 1:19); ν_{max} (film) 2955 (w), 1736 (s), 1636 (m), 1538 (s), 1483 (m), 1366 (m), 1296 (m), 1229 (s), 1202 (s), 1169 (m), 1042 (m), 969 (m), 933 (m) cm^{-1}; δ_H (300 MHz, $CDCl_3$) 5.05 (1H,

s, C=C*H*), 4.10 (2H, t, *J* 6.2 Hz, CH_2OAc), 3.39 (2H, t, *J* 7.2 Hz, CH_2N), 3.31 (2H, t, *J* 7.2 Hz, CH_2N), 3.23 (2H, t, *J* 7.8 Hz, CH_2C=), 2.09 and 2.06 (2 × 3H, 2 × s, =CHCOCH_3 and OCOCH_3), 1.96 and 1.93 (4H, overlapping quintets, *J* 7.3 and 6.3 Hz, 2 × $CH_2CH_2CH_2$); δ_C (75 MHz, $CDCl_3$) 194.1, 170.8, 165.1, 89.6, 61.8, 52.5, 43.1, 33.4, 30.7, 25.5, 21.0. HRMS (EI) found, 225.1356. $C_{12}H_{19}NO_3$ requires 225.1359.

*Ethyl (2E)-{1-[3-(acetoxy)propyl]-2-pyrrolidinylidene}ethanoate (**7b**)*

A solution of 3-(2-thioxo-1-pyrrolidinyl)propyl acetate (**3**, 3.89 g, 19.3 mmol) and ethyl bromoacetate (3.91 g, 2.25 mL, 20.3 mmol) were allowed to react in dry CH_2Cl_2 (40 mL) followed by treatment with PPh_3 (5.33 g, 20.3 mmol) and NEt_3 (2.05 g, 2.83 mL, 20.3 mmol) in MeCN (61 mL) according to the general procedure to afford (2*E*)-{1-[3-(acetoxy)propyl]-2-pyrrolidinylidene}ethanoate (**7b**) as a light yellow oil (4.18 g, 90%); R_f 0.44 (EtOAc:Hex 1:1); ν_{max} (film) 2972 (w), 1736 (s), 1680 (m), 1586 (s), 1462 (w), 1427 (m), 1367 (w), 1230 (s), 1134 (s), 1052 (s), 958 (w), 858 (w), 783 (m) cm^{-1}; δ_H (300 MHz, $CDCl_3$) 4.53 (1H, s, =C*H*), 4.10 (2H, q, *J* 7.2 Hz, OCH_2CH$_3$), 4.07 (2H, t, *J* 6.1 Hz, CH_2OAc), 3.37 (2H, t, *J* 7.1 Hz, CH_2N), 3.27 (2H, t, *J* 7.2 Hz, CH_2N), 3.16 (2H, t, *J* 7.8 Hz, CH_2C=), 2.08 (3H, s, OCOCH_3), 1.95 and 1.92 (4H, 2 × overlapping quintets, *J* 7.5 and 6.8 Hz, 2 × $CH_2CH_2CH_2$), 1.25 (3H, t, *J* 7.1 Hz, OCH$_2$CH_3); δ_C (75 MHz, $CDCl_3$) 171.0, 169.5, 164.9, 78.1, 61.9, 58.3, 52.8, 43.1, 32.7, 25.5, 21.2, 21.0, 14.8; m/z (EI) 255 (27), 43 (24), 97 (21), 168 (44), 169 (42), 196 (100), 210 (47), 212 (21), 255 (27). HRMS (EI) found, 255.1465. $C_{13}H_{21}NO_4$ requires 255.1465.

*3-[(2E)-2-(Cyanomethylene)pyrrolidinyl]propyl acetate (**7c**)*

3-(2-Thioxo-1-pyrrolidinyl)propyl acetate (**3**, 1.01 g, 5.00 mmol) and bromoacetonitrile (0.630 g, 0.370 mL, 5.25 mmol) were allowed to react in dry CH_2Cl_2 (10 mL) followed by treatment with PPh_3 (1.38 g, 5.25 mmol) and NEt_3 (0.531 g, 5.25 mmol) in MeCN (15 mL) according to the general procedure to afford 3-[(2*E*)-2-(cyanomethylene)pyrrolidinyl]propyl acetate (**7c**) as a light yellow oil (0.462 g, 44%); R_f 0.69 (EtOAc); ν_{max} (film) 3070 (w), 2963 (w), 2874 (w), 2187 (m), 1734 (s), 1600 (s), 1460 (w), 1429 (m), 1336 (m), 1293 (m), 1229 (s), 1039 (m), 936 (w), 863 (w), 801 (w), 694 (m) cm^{-1}; δ_H (300 MHz, $CDCl_3$) 4.07 (2H, t, *J* 6.2 Hz, CH_2OAc), 3.67 (1H, s, C=C*H*), 3.45 (2H, t, *J* 6.9 Hz, CH_2N), 3.20 (2H, t, *J* 7.1 Hz, CH_2N), 2.88 (2H, t, *J* 7.6 Hz, CH_2C=), 2.08 (3H, s, $OCOCH_3$), 2.00 and 1.90 (2 × 2H, 2 × quintets, *J* 7.5 and 6.7 Hz, 2 × $CH_2CH_2CH_2$); δ_C (75 MHz, $CDCl_3$) 170.9, 165.6, 122.7, 61.6, 53.8, 53.7, 43.1, 32.8, 25.5, 20.9, 20.9. HRMS (EI) found, 208.1228. $C_{11}H_{16}N_2O_2$ requires 208.1206.

General procedure for acetate hydrolysis

To a stirred solution of the required enaminone **7** in MeOH (3.6 mL $mmol^{-1}$) was added K_2CO_3 (1.1–2.0 equiv). After 3 h the mixture was filtered through celite. The filtrate was evaporated in vacuo, and then taken up in $CHCl_3$ (10 mL $mmol^{-1}$) and washed with satd. aq. NaCl solution (10 mL $mmol^{-1}$). The aqueous phases were back extracted with $CHCl_3$ (3 × 10 mL $mmol^{-1}$), dried ($MgSO_4$) filtered and evaporated in vacuo to afford the crude product. The crude mixture was purified by column chromatography to yield the desired alcohols **8**.

*(1E)-1-[1-(3-Hydroxypropyl)-2-pyrrolidinylidene]-2-propanone (**8a**)*

3-[(2*E*)-2-(2-Oxopropylidene)pyrrolidinyl]propyl acetate (**7a**, 0.792 g, 3.51 mmol) and K_2CO_3 (0.534 g, 3.86 mmol) in MeOH (13 mL) were allowed to react according to the general procedure to yield (1*E*)-1-[1-(3-hydroxypropyl)-2-pyrrolidinylidene]-2-propanone (**8a**, 0.527 g, 82%) as a yellow oil; R_f 0.22 (CH_3OH:CH_2Cl_2 1:19); ν_{max} (film) 3366 (v br. w), 2927 (w), 2872 (w), 1732 (m), 1630 (m), 1568 (m), 1427 (m), 1367 (m), 1236 (s), 1047 (m) cm^{-1}; δ_H (300 MHz, $CDCl_3$) 5.10 (1H, s, C=C*H*), 3.68 (2H, t, *J* 6.0 Hz, C*H₂*OH), 3.42 (2H, t, *J* 7.3 Hz, CH_2N), 3.36 (2H, t, *J* 7.1 Hz, CH_2N), 3.21 (2H, t, *J* 7.8 Hz, CH_2C=), 2.30 (1H, br s, O*H*), 2.05 (3H, s, COCH_3), 1.94 and 1.84 (2 × 2H, 2 × quintets, *J* 7.6 and 6.6 Hz, 2 × $CH_2CH_2CH_2$); δ_C (75 MHz, $CDCl_3$) 194.5, 165.8, 89.5, 59.9, 52.8, 43.4, 33.7, 30.6, 29.2, 21.0. HRMS (EI) found, 183.1253. $C_{10}H_{17}NO_2$ requires 183.1254.

*Ethyl (2E)-[1-(3-hydroxypropyl)-2-pyrrolidinylidene]ethanoate (**8b**)*

Ethyl (2*E*)-{1-[3-(acetoxy)propyl]-2-pyrrolidinylidene}ethanoate (**7b**, 4.19 g, 17.6 mmol) and K_2CO_3 (2.68 g, 19.3 mmol) in MeOH (63 mL) were allowed to react according to the general procedure to yield ethyl (2*E*)-[1-(3-hydroxypropyl)-2-pyrrolidinylidene]ethanoate (**8b**, 3.19 g, 85%) as a yellow oil; R_f 0.18 (EtOAc:Hex 1:1); ν_{max} (film) 3415 (v br, w), 2971 (w), 2940 (w), 2872 (w), 1727 (m), 1657 (m), 1579 (s), 1462 (w), 1376 (w), 1294 (m), 1248 (m), 1202 (m), 1132 (s), 1052 (s), 782 (m) cm^{-1}; δ_H (300 MHz, $CDCl_3$) 4.56 (1H, s, C=C*H*), 4.07 (2H, q, *J* 7.2 Hz, OCH_2CH₃), 3.67 (2H, t, *J* 6.1 Hz, CH_2OH), 3.39 (2H, t, *J* 7.1 Hz, CH_2N), 3.31(2H, t, *J* 7.1 Hz, CH_2N), 3.15 (2H, t, *J* 7.8 Hz, CH_2C=), 1.99 (1H, br s, O*H*), 1.94 and 1.82 (2 × 2H, 2 × quintets, *J* 7.5 and 6.6 Hz, 2 × $CH_2CH_2CH_2$), 1.25 (3H, t, *J* 7.1 Hz, OCH_2CH_3); δ_C (75 MHz, $CDCl_3$) 169.8, 165.2, 77.7, 60.2, 58.4, 52.9, 43.2, 32.9, 29.1, 21.2, 14.9. HRMS

(EI) found, 13.1369. $C_{11}H_{19}NO_3$ requires 213.1359. The data agree with those reported for the product prepared by alternative methods [3, 4].

*(2E)-[1-(3-Hydroxypropyl)-2-pyrrolidinylidene]ethanenitrile (**8c**)*

3-[(2*E*)-2-(Cyanomethylene)pyrrolidinyl]propyl acetate (**8c**, 1.37 g, 6.56 mmol) and K_2CO_3 (1.31 g, 13.1 mmol) in MeOH (24 mL) were allowed to react according to the general procedure to yield (2*E*)-[1-(3-hydroxypropyl)-2-pyrrolidinylidene]ethanenitrile (**8c**, 0.972 g, 5.85 mmol, 89%) as a yellow oil; R_f 0.41 (CH_3OH:CH_2Cl_2 1:19); ν_{max} (film) 3403 (v br, w), 3071 (w), 2942 (w), 2873 (w), 2178 (m), 1595 (s), 1460 (w), 1429 (m), 1289 (m), 1153 (w), 1052 (m), 689 (m) cm^{-1}; δ_H (300 MHz, $CDCl_3$) 3.73 (1H, s, =C*H*), 3.64 (2H, t, *J* 5.9 Hz, CH_2OH), 3.47 (2H, t, *J* 6.9 Hz, CH_2N), 3.25 (2H, t, *J* 7.1 Hz, CH_2N), 2.86 (2H, t, *J* 7.8 Hz, CH_2C=), 2.47 (1H, br s, O*H*), 1.99 and 1.79 (2 × 2H, 2 × quintets, *J* 7.3 and 6.5 Hz, 2 × $CH_2CH_2CH_2$); δ_C (75 MHz, $CDCl_3$) 165.9, 123.4, 59.6, 53.8, 52.9, 43.1, 32.9, 29.0, 20.9. HRMS (EI) found, 166.1094. $C_9H_{14}N_2O$ requires 166.1101.

General procedure for the alkylative ring closure to 1,2,3,5,6,7-hexahydroindolizines

A stirring solution of alcohol **8** in a mixture of MeCN (6.2 mL $mmol^{-1}$) and PhMe (3.1 mL $mmol^{-1}$) was charged with PPh_3 (2.0–3.0 equiv) and imidazole (2.0–3.0 equiv). Once the solids had dissolved, I_2 (2.0 equiv) was added in one portion. The homogeneous solution was stirred under reflux for 1 h. The reaction was quenched by the addition of a solution of satd. aq. $NaHCO_3$ (10 mL $mmol^{-1}$), and the aqueous residue was extracted with EtOAc (3 × 10 mL $mmol^{-1}$). The combined organic fractions were washed with satd. aq. $Na_2S_2O_3$ solution (10 mL $mmol^{-1}$). The organic washings were dried ($MgSO_4$), filtered and evaporated in vacuo to yield the

crude product. Purification by column chromatography on silica gel yielded the desired bicyclic compounds **9**.

*1-(1,2,3,5,6,7-Hexahydroindolizin-8-yl)ethanone (**9a**)*

(1*E*)-1-[1-(3-Hydroxypropyl)-2-pyrrolidinylidene]-2-propanone (**8a**, 2.35 g, 12.9 mmol), PPh_3 (10.1 g, 38.5 mmol, 3.0 equiv) and imidazole (2.63 g, 38.5 mmol) in MeCN (80 mL) and PhMe (40 mL) followed by I_2 (6.50 g, 25.7 mmol) were allowed to react according to the general procedure to yield 1-(1,2,3,5,6,7-hexahydro-8-indolizinyl)ethanone (**9a**, 0.567 g, 27%) as a clear oil; R_f 0.32 (CH_3OH:CH_2Cl_2 1:19); δ_H (300 MHz, $CDCl_3$) 7.60-7.34 (PPh_3 residues), 3.26 (2H, td, *J* 7.2 and 1.8 Hz, CH_2N), 3.11 and 3.05 (4H, overlapping t, *J* 5.6 and 6.8 Hz, CH_2N and $CH_2C(COCH_3)=C$), 2.33 (2H, t *J* 6.0 Hz, $CH_2C=C(COCH_3)$), 2.03 (3H, s, $COCH_3$), 1.84-1.70 (4H, m, remaining CH_2). Complete removal of phosphine residues was not successful.

*Ethyl 1,2,3,5,6,7-hexahydroindolizine-8-carboxylate (**9b**)*

Ethyl (2*E*)-[1-(3-hydroxypropyl)-2-pyrrolidinylidene]ethanoate (**8b**, 0.865 g, 4.05 mmol), PPh_3 (3.19 g, 12.2 mmol, 3.0 equiv) and imidazole (0.827 g, 12.2 mmol, 3.0 equiv) in MeCN (26 mL) and PhMe (13 mL) followed by I_2 (2.06 g, 8.10 mmol) were allowed to react according to the general procedure to yield ethyl 1,2,3,5,6,7-hexahydro-8-indolizinecarboxylate (**9b**, 0.438 g, 59%) as a clear oil; R_f 0.61 (EtOAc:Hex 1:1); ν_{max} (film) 2943 (w), 2845 (w), 1674 (m), 1584 (s), 1425 (w), 1368 (m), 1283 (m), 1255 (s), 1215 (m), 1181 (m), 1150 (s), 1095 (m), 1041 (w), 882 (w) 852 (w) 763 (m) cm–1; δ_H (300 MHz, $CDCl_3$) 4.00 (2H, q, *J* 7.1 Hz, OCH_2CH_3), 3.19 (2H, t, *J* 7.0 Hz, CH_2N), 3.06 (2H, t, *J* 5.7 Hz, CH_2N), 2.96 (2H, t, *J* 7.8 Hz,

$CH_2C(CO_2Et)$=C), 2.25 (2H, t, *J* 6.3 Hz, CH_2C=CCO_2Et), 1.82 and 1.73 (2 × 2H, 2 × quintets, *J* 7.4 and 6.0 Hz, remaining CH_2), 1.16 (3H, t, *J* 7.2 Hz, OCH_2CH_3); δ_C (75 MHz, $CDCl_3$) 168.3, 158.7, 87.1, 57.9, 52.6, 44.6, 32.3, 21.2, 21.1, 20.6, 14.5. HRMS (EI) found, 195.1247. $C_{11}H_{17}NO_2$ requires 195.1254. The NMR spectroscopic data agree with those reported by Kim et al. [5].

*1,2,3,5,6,7-Hexahydroindolizine-8-carbonitrile (**9c**)*

(2*E*)-[1-(3-Hydroxypropyl)-2-pyrrolidinylidene]ethanenitrile (**8c**, 0.583 g, 0.519 g, 3.51 mmol), PPh_3 (1.84 g, 7.02 mmol, 2.0 equiv) and imidazole (0.479 g, 7.02 mmol, 2.0 equiv) in MeCN (21 mL) and PhMe (11 mL) followed by I_2 (1.76 g, 7.02 mmol) were allowed to react according to the general procedure to yield *1,2,3,5,6,-7-hexahydro-indolizine-8-carbonitrile* (**9c**) as a clear oil (0.375 g, 72%); R_f 0.75 (MeOH:CH_2Cl_2 1:19); ν_{max} (film) 2930 (w), 2849 (w), 2173 (m), 1615 (s), 1428 (m), 1361 (m), 1289 (s), 1212 (m), 1182 (m), 1149 (m), 1108 (m), 1081 (m) cm–1; δ_H (300 MHz, $CDCl_3$) 3.32 (2H, t, *J* 6.8 Hz, CH_2N), 3.15 (2H, t, *J* 5.6 Hz, CH_2N), 2.74 (2H, t, *J* 7.7 Hz, CH_2C(CN)=C), 2.23 (2H, t, *J* 6.1 Hz, CH_2C=CCN), 1.97 and 1.84 (2 × 2H, 2 × quintets, *J* 7.3 and 5.9 Hz, remaining CH_2); δ_C (75 MHz, $CDCl_3$) 159.4, 124.0, 64.4, 53.4, 44.2, 30.7, 22.2, 21.1, 20.8. HRMS (EI) found, 148.1000. $C_9H_{12}N_2$ requires 148.0995.

*General procedure for the tosylation of alcohols **8***

To a solution of *p*-toluenesulfonyl chloride (1.4 equiv) in CH_2Cl_2 (9 mL $mmol^{-1}$) at rt was added NEt_3 (9.8 equiv) and DMAP (0.1 equiv). After 30 min the alcohol **8** was added in one portion. The solution turned brown over time and after 18 h the solution was washed with H_2O (10 mL $mmol^{-1}$). The organic layer was separated, dried

($MgSO_4$), filtered and evaporated in vacuo to yield a brown solid. The crude solid was purified by column chromatography on silica gel to yield the desired products.

*3-[(2E)-2-(Cyanomethylene)pyrrolidinyl]propyl 4-methylbenzenesulfonate (**10c**) and (2E)-[1-(3-Chloropropyl)-2-pyrrolidinylidene]ethane-nitrile (**11c**)*

(2*E*)-[1-(3-Hydroxypropyl)-2-pyrrolidinylidene]ethanenitrile (**8c**, 0.694 g, 4.18 mmol), *p*-TsCl (1.15 g, 5.85 mmol), NEt_3 (4.14 g, 5.71 mL, 40.9 mmol) and DMAP (0.055 g, 0.418 mmol) in CH_2Cl_2 (38 mL) were allowed to react according to the general procedure to yield 3-[(2*E*)-2-(cyanomethylene)pyrrolidinyl]propyl-4-methyl-benzenesulfonate (**10c**, 0.261 g, 19%) as a yellow solid and (2*E*)-[1-(3-chloropropyl)-2-pyrrolidinylidene]ethanenitrile (**11c**, trace) as a brown oil.

Compound **10c**: R_f 0.17 (EtOAc:Hex 1:1); ν_{max} (film) 3058 (w), 2967 (w), 2941 (w), 2891 (w), 2178 (m), 1599 (w), 1493 (s), 1377 (m), 1359 (s), 1311 (m), 1293 (m), 1187 (m), 1171 (s), 1095 (m), 1019 (m), 959 (m), 919 (s), 828 (s), 810 (s), 721 (s), 661 (s) cm^{-1}; δ_H (300 MHz, $CDCl_3$) 7.79 (2H, d, *J* 8.2 Hz, Ar*H*), 7.38 (2H, d, *J* 8.0 Hz, Ar*H*), 4.04 (2H, t, *J* 5.8 Hz, C*H₂*OTs), 3.55 (1H, s, C=C*H*), 3.38 (2H, t, *J* 6.9 Hz, C*H₂*N), 3.18 (2H, t, *J* 6.9 Hz, C*H₂*N), 2.81 (2H, t, *J* 7.7 Hz, C*H₂*C=), 2.47 (3H, s, ArC*H₃*), 1.92-1.91 (4H, m, remaining C*H₂*); δ_C (75 MHz, $CDCl_3$) 165.5, 145.3, 132.7, 131.1, 127.9, 122.5, 67.5, 54.2, 53.9, 42.6, 32.8, 25.8, 21.8, 20.9.

Compound **11c**: R_f 0.31 (EtOAc:Hex 1:1); ν_{max} (film) 2963 (w), 2868 (w), 2187 (m), 1598 (s), 1428 (m), 1361 (w), 1272 (m), 1143 (w), 695 (m), 652 (w) cm^{-1}; δ_H (300 MHz, $CDCl_3$) 3.73 (1H, s, C=C*H*), 3.55 (2H, t, *J* 6.1 Hz, C*H₂*Cl), 3.47 (2H, t, *J* 6.9 Hz, C*H₂*N), 3.30 (2H, t, *J* 6.9 Hz, C*H₂*N), 2.88 (2H, t, *J* 7.8 Hz, C*H₂*C=), 2.03 and 2.00 (4H, overlapping quintets, *J* 6.2 and 7.3 Hz, remaining C*H₂*); δ_C (300 MHz, $CDCl_3$)

165.7, 122.6, 54.1 (2 signals), 43.5, 42.2, 32.8, 29.0, 21.0. HRMS (EI) found, 184.0762. $C_9H_{13}ClN_2$ requires 184.0762.

*3-((2E)-2-{2-[Methoxy(methyl)amino]-2-oxoethylidene}-pyrrolidinyl)propyl 4-methyl-benzenesulfonate (**10d**) and (2E)-2-[1-(3-chloro-propyl)-2-pyrrolidinylidene]-N-methoxy-N-methylethanamide (**11d**)*

(2*E*)-2-[1-(3-Hydroxypropyl)-2-pyrrolidinylidene]-*N*-methoxy-*N*-methylethanamide (**8d**, 0.202 g, 0.896 mmol), *p*-TsCl (0.245 g, 1.25 mmol, 1.4 equiv), NEt_3 (0.889 g, 1.2 mL 8.78 mmol) and DMAP (11.0 mg, 0.09 mmol) in CH_2Cl_2 (7.8 mL) were allowed to react according to the general procedure to yield 3-((2*E*)-2-{2-[methoxy(methyl)amino]-2-oxoethylidene}-pyrrolidinyl)propyl 4-methylbenzenesulfonate (**10d**, 0.204 g, 0.639 mmol, 71%) as a brown oil containing trace amounts of (2*E*)-2-[1-(3-chloropropyl)-2-pyrrolidinylidene]-*N*-methoxy-*N*-methylethanamide (**11d**).

Compound **10d**: R_f 0.37 (EtOAc:Hex 1:1); ν_{max} (film) 3450 (w), 2942 (w), 1652 (s), 1493 (m), 1447 (m), 1414 (m), 1172 (s), 1119 (s), 1032 (s), 1010 (s), 817 (m), 680 (s) cm^{-1}; δ_H (300 MHz, $CDCl_3$) 7.79 (2H, d, *J* 8.1 Hz, Ar*H*), 7.36 (2H, d, *J* 8.0 Hz, Ar*H*), 5.04 (1H, s, =C*H*), 4.05 (2H, t, *J* 5.9 Hz, C*H₂*OTs), 3.65 (3H, s, OC*H₃*), 3.29 and 3.28 (4H, overlapping t, *J* 7.0 and 7.0 Hz, 2 × C*H₂*N), 3.21–3.17 (2H, m, C*H₂*C=), 3.14 (3H, s, NC*H₃*), 2.45 (3H, s, ArC*H₃*), 1.95 and 1.88 (2 × 2H, 2 × quintets, *J* 6.6 and 7.5 Hz, remaining C*H₂*); δ_C (75 MHz, $CDCl_3$) 171.8, 164.3, 145.1, 130.0, 128.8, 125.0, 77.2, 67.9, 61.1, 52.7, 42.6, 33.1, 32.6, 25.8, 21.7, 21.4.

Identifiable peaks for (2*E*)-2-[1-(3-chloropropyl)-2-pyrrolidinylidene]-*N*-methoxy-*N*-methylethanamide (**11d**): δ_H (300 MHz, $CDCl_3$) 5.16 (s, =C*H*), 3.68 (s, OC*H₃*), 3.45–3.34 (m, 2 × C*H₂*N and CH_2C=), 3.17 (s, NC*H₃*), 2.11–2.00 (m, remaining C*H₂*).

*General procedure for the catalytic hydrogenation of 1,2,3,5,6,7-hexahydroindolizines **9***

To a solution of the bicyclic enamine **9** in glacial acetic acid (5.5 mL $mmol^{-1}$) was added Adams' catalyst (5×10^{-2} g $mmol^{-1}$) and the mixture was stirred under a hydrogen atmosphere (1 atm) for 24 h. The mixture was filtered through celite and washed copiously with EtOH, after which the solvent was evaporated in vacuo to yield the crude products. Purification by column chromatography on silica gel yielded the desired reduced compounds **12**.

Ethyl (8R,8aR*)-octahydroindolizine-8-carboxylate (**12b'**) and ethyl (8R*,8aS*)-octahydroindolizine-8-carboxylate (**12b"**)*

Ethyl 1,2,3,5,6,7-hexahydroindolizine-8-carboxylate (**9b**, 0.513 g, 2.63 mmol) and Adams' catalyst (0.132 g) in glacial acetic acid (14.5 mL) were allowed to react according to the general procedure to yield a mixture of the diastereomers ethyl (8*R**,8a*R**)-octahydroindolizine-8-carboxylate (**12b'**) and ethyl (8*R**,8a*S**)-octahydroindolizine-8-carboxylate (**12b"**) (0.375 g, 72%; dr 85:15) as a clear oil. The mixture was partially separated by flash column chromatography (5% MeOH/CH_2Cl_2), affording enriched samples of **12b'** and **12b"** for characterisation. Their identities were confirmed by comparison of the spectra with those reported by Kiss et al. [6].

Isomer **12b'**: R_f 0.29 (MeOH:CH_2Cl_2 1:19); ν_{max} (film) 3402, 2940 (w), 1727 (s), 1660 (m), 1587 (m), 1445 (m), 1369 (m), 1302 (m), 1259 (m), 1182 (m), 1156 (m), 1107 (m), 1022 (m) cm^{-1}; δ_H (300 MHz, $CDCl_3$) 4.16-3.99 (2H, m, OCH_2CH_3), 3.04-2.96 (2H, m), 2.71-2.70 (1H, m), 2.14-2.07 (1H, m), 2.05-1.88 (4H, m), 1.83-1.33 (6H, m), 1.19(3H, t, *J* 7.1 Hz, OCH_2CH_3); δ_C (75 MHz, $CDCl_3$) 173.1, 64.5, 59.8, 54.8, 53.0,

41.7, 26.6, 26.2, 22.4, 20.6, 14.3. HRMS (EI) found, 197.1418. $C_{11}H_{19}NO_2$ requires 197.1410.

Isomer **12b"**: R_f 0.36 ($MeOH:CH_2Cl_2$ 1:19); ν_{max} (film) 3420, 2932 (w), 2851 (w), 1726 (s), 1665 (s), 1419 (w), 1293 (w), 1192 (m), 1173 (s), 1119 (m), 1026 (m) cm^{-1}; δ_H (300 MHz, $CDCl_3$) 4.13 (2H, q, *J* 7.1 Hz, OCH_2CH_3), 3.06 (2H, td, *J* 8.8 and 2.0 Hz), 2.26-2.22 (1H, m), 2.13 (1H, q, *J* 9.0 Hz), 2.06-1.90 (4H, m), 1.86–1.56 (4H, m), 1.55-1.37 (2H, m), 1.26 (3H, t, *J* 7.1 Hz, CH_2CH_3); δ_C (300 MHz, $CDCl_3$) 174.4, 65.2, 60.3, 54.1, 52.4, 48.3, 29.3, 28.2, 24.9, 20.6, 14.4. HRMS (EI) found, 197.1396. $C_{11}H_{19}NO_2$ requires 197.1410.

*Octahydroindolizine-8-carbonitrile (**12c**)*

1,2,3,5,6,7-Hexahydroindolizine-8-carbonitrile (**9c**, 0.472 g, 3.19 mmol) and Adams' catalyst (0.160 g) in glacial acetic acid (17.5 mL) were allowed to react according to the general procedure to yield an inseparable mixture of the (8*R**,*8aR**)- and (8*R**,8a*S**)-diastereomers of octahydroindolizine-8-carbonitrile (**12c**) in a ratio of 92:8 as an orange oil (0.629 g, 85%); R_f 0.13 ($MeOH:CH_2Cl_2$ 1:19); ν_{max} (film) 2955 (w), 2923 (m), 2854 (w), 2360 (w), 1728 (w), 1658 (w), 1456 (m), 1260 (m), 1092 (m), 1062 (m), 1029 (m), 800 (m) cm^{-1}; δ_H (300 MHz, $CDCl_3$) 3.16–3.02 (2H, m), 2.96–2.95 (1H, m), 2.16–1.58 (*ca* 10H, m), 1.55–1.42 (<2H, m); δ_C (75 MHz, $CDCl_3$) major isomer 120.1, 63.4, 54.0, 52.2, 32.1, 28.5, 27.7, 22.2, 20.5; δ_C (75 MHz, $CDCl_3$) minor isomer 120.8, 65.1, 54.1, 51.7, 33.4, 29.6, 28.7, 24.4, 20.2.

*(±)-Tashiromine (**1**) and (±)-epitashiromine (**2**)*

The diastereomeric mixture of ethyl (8*R**,8a*R**)-octahydroindolizine-8-carboxylate (**12b'**) and ethyl (8*R**,8a*S**)-octahydroindolizine-8-carboxylate (**12b"**) (0.675 g,

3.42 mmol; dr 85:15) in Et_2O (13.7 mL) was added dropwise to a slurry of $LiAlH_4$ (0.196 g, 5.13 mmol) in Et_2O (23 mL) at 0 °C. The mixture was warmed to rt and stirred for a further 16 h. The reaction was quenched by the sequential addition of H_2O (0.8 mL), aq. NaOH (0.8 mL, 15% w/v) and finally H_2O (2.4 mL). The solids were removed by passing the mixture through a thin pad of celite. The filtrate was dried (anhydrous Na_2SO_4), filtered and evaporated in vacuo to yield (±)-epitashiromine (**2**) and (±)-tashiromine (**1**) in the ratio 87:13 (0.464 g, 87%). The two diastereomers were partially separated by flash column chromatography for characterisation using $MeOH/CH_2Cl_2/NH_4OH$ 95:4.75:0.25 as eluent.

(±)-Tashiromine (**1**): Yellow oil; δ_H (300 MHz, $CDCl_3$) 3.60 (1H, dd, *J* 10.7 and 4.6 Hz, CH_aH_bOH), 3.43 (1H, dd, *J* 10.7 and 6.6 Hz, CH_aH_bOH), 3.25(1H, br s, O*H*), 3.12–3.04 (2H, m, H-4_{eq} & H-5_{eq}), 2.08 (1H, q, *J* 9.1 Hz), 1.98–1.85, 1.98 and 1.90 (3H, overlapping m and 2 × dd, *J* 11.4 and 3.2 Hz, and 13.3 and 3.5 Hz), 1.85–1.59 (4H, m), 1.55–1.42 (2H, m), 1.04 (2H, qd, *J* 12.3 and 4.9 Hz); δ_C (75 MHz, $CDCl_3$) 66.6, 65.2, 54.1, 52.7, 44.5, 29.0, 27.7, 25.0, 20.7. HRMS (EI) found, 155.1294. $C_9H_{17}NO$ requires 155.1310.

(±)-Epitashiromine (**2**): Yellow oil; δ_H (300 MHz, $CDCl_3$) 4.9–4.2 (1H, br s, O*H*), 4.12 (1H, dd, *J* 10.7 and 4.4 Hz, CH_aH_bOH), 3.74 (1H, dd, *J* 10.7 and 1.6 Hz, CH_aH_bOH), 3.09 (1H, br dd, *J ca* 6.4 and 2.5 Hz), 3.01 (1H, ddd, *J* 9.1, 2.9 and 1.8 Hz, H-5_{eq}), 2.29–2.23 (1H, m), 2.07–1.95 (3H, m), 1.93–1.87 (2H, m), 1.84–1.65 (4H, m), 1.64–1.47 (2H, m). δ_C (75 MHz, $CDCl_3$) 66.8, 65.4, 54.5, 53.6, 35.5, 30.4, 25.9, 23.2, 20.8. HRMS (EI) found, 155.12965. $C_9H_{17}NO$ requires 155.1310.

References

1. Perrin, D. D.; Armarego, W. L. F. In *Purification of Laboratory Chemicals*, Third Edition; Pergamon Press: Oxford; 1988, 291.

2. Michael, J. P.; Parsons, A. S.; *S. Afr. J. Chem.*, **1993**, *46*, 65–69.

3. Michael, J. P.; de Koning, C. B.; San Fat, C.; Nattrass, G. L. *ARKIVOC* **2002**, (ix), 62–77.

4. Michael, J. P.; de Koning, C. B.; Malefetse, T. J.; Yillah, I. *Org. Biomol. Chem.* **2004**, *2*, 3510–3517.

5. Kim, J. H.; Shin, H.; Lee, S. *J. Org. Chem.* **2012**, *77*, 1560–1575.

6. Kiss, L.; Forró, E.; Fülöp, F. *Beilstein J. Org. Chem.* **2015**, *11*, 596–603.

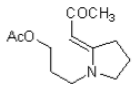

3-[(2*E*)-2-(2-Oxopropylidene)pyrrolidinyl]propyl acetate (**7a**)

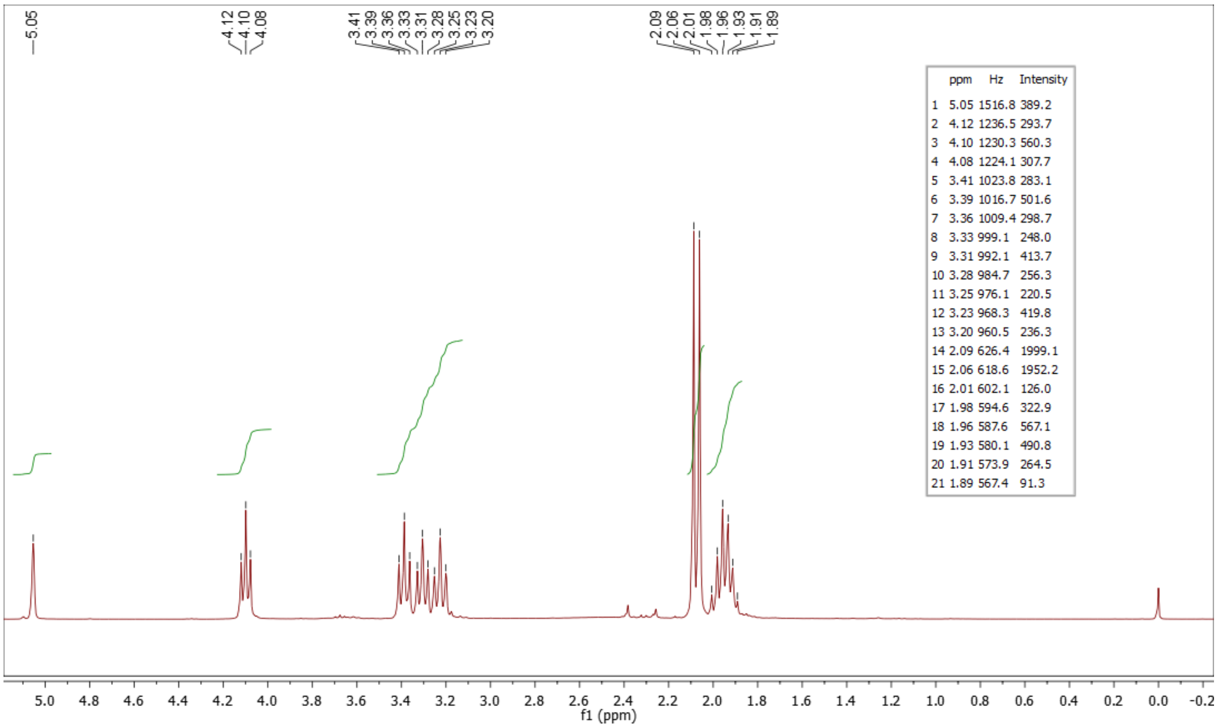

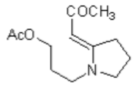

3-[(2*E*)-2-(2-Oxopropylidene)pyrrolidinyl]propyl acetate (**7a**)

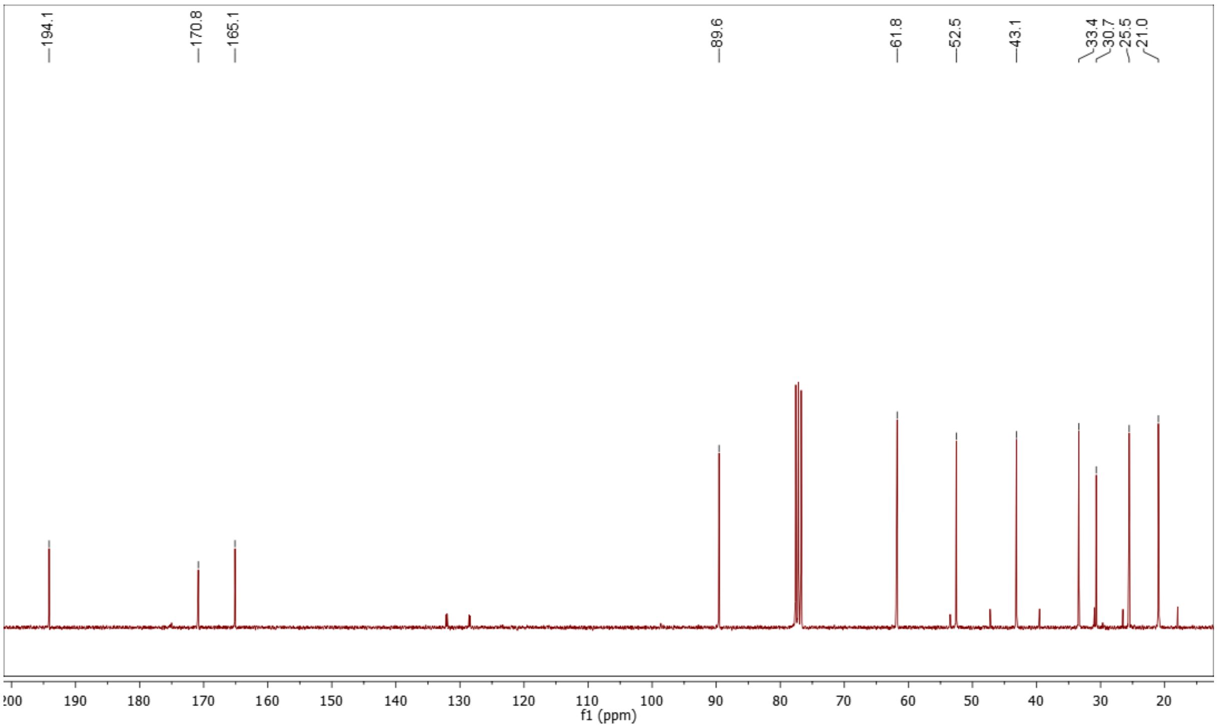

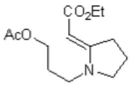

Ethyl (2*E*)-{1-[3-(acetoxy)propyl]-2-pyrrolidinylidene}ethanoate (**7b**)

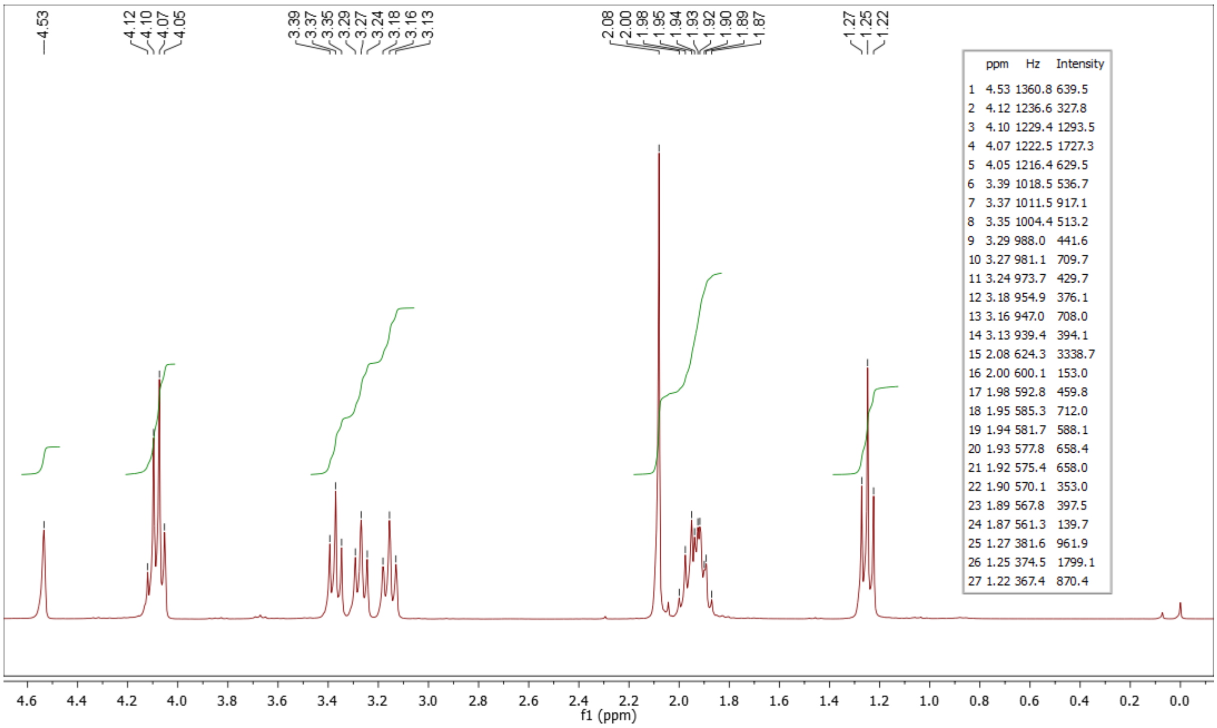

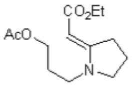

Ethyl (2*E*)-{1-[3-(acetoxy)propyl]-2-pyrrolidinylidene}ethanoate (**7b**)

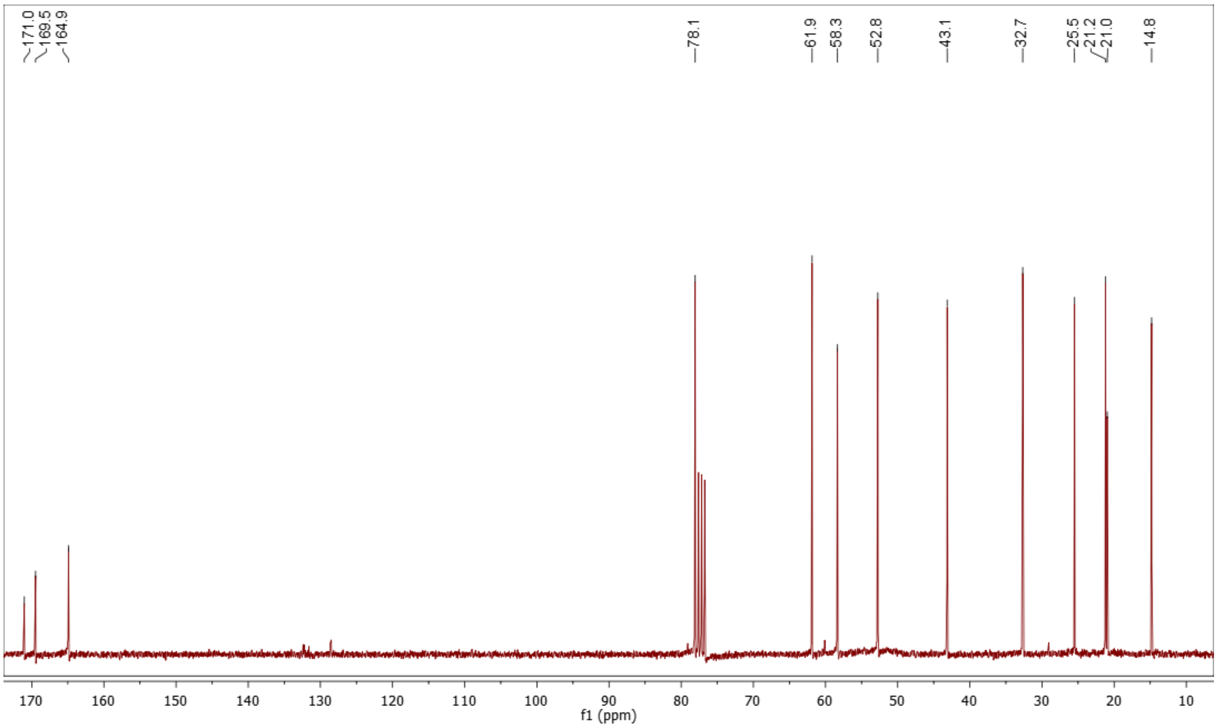

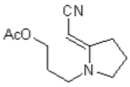

3-[(2*E*)-2-(Cyanomethylene)pyrrolidinyl]propyl acetate (**7c**)

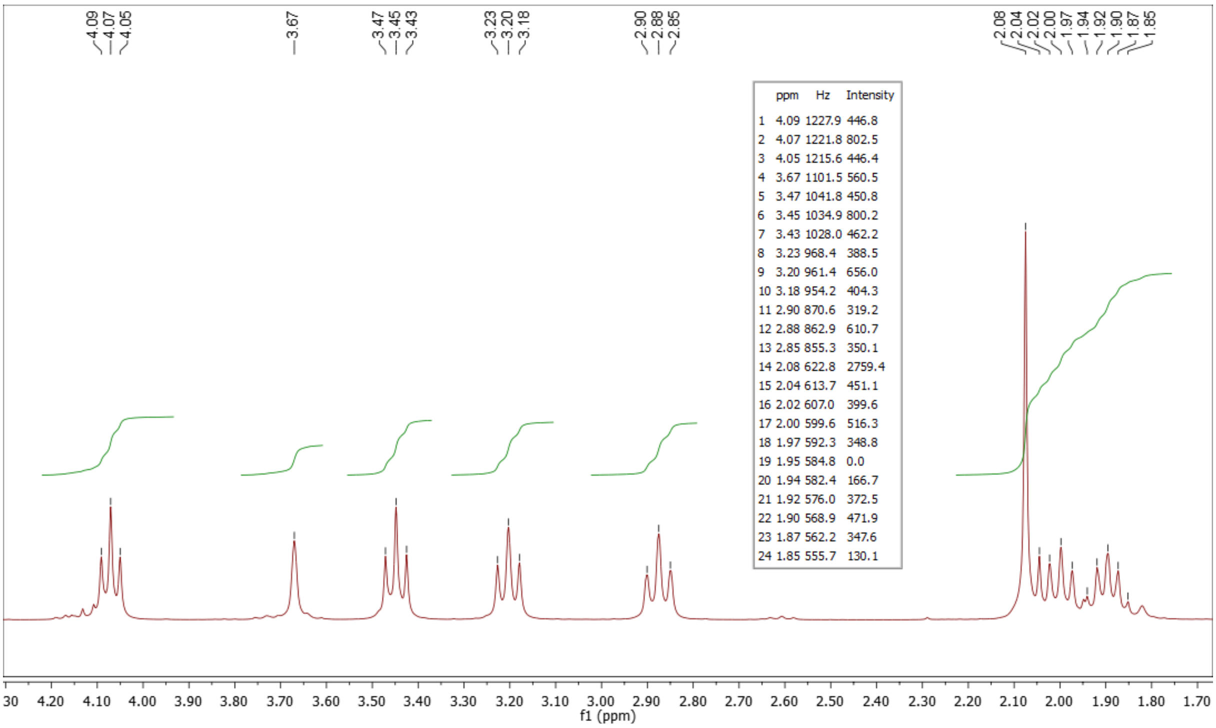

	ppm	Hz	Intensity
1	4.09	1227.9	446.8
2	4.07	1221.8	802.5
3	4.05	1215.6	446.4
4	3.67	1101.5	560.5
5	3.47	1041.8	450.8
6	3.45	1034.9	800.2
7	3.43	1028.0	462.2
8	3.23	968.4	388.5
9	3.20	961.4	656.0
10	3.18	954.2	404.3
11	2.90	870.6	319.2
12	2.88	862.9	610.7
13	2.85	855.3	350.1
14	2.08	622.8	2759.4
15	2.04	613.7	451.1
16	2.02	607.0	399.6
17	2.00	599.6	516.3
18	1.97	592.3	348.8
19	1.95	584.8	0.0
20	1.94	582.4	166.7
21	1.92	576.0	372.5
22	1.90	568.9	471.9
23	1.87	562.2	347.6
24	1.85	555.7	130.1

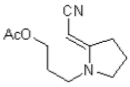

3-[(2*E*)-2-(Cyanomethylene)pyrrolidinyl]propyl acetate (**7c**)

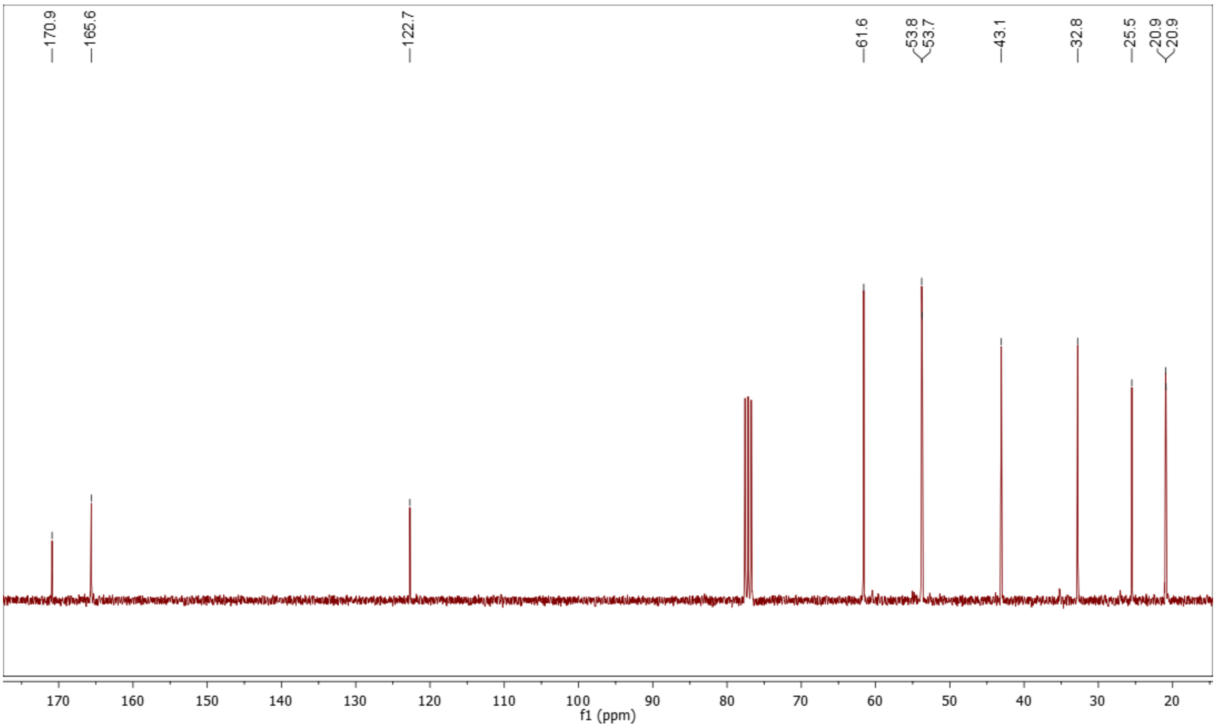

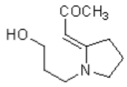

(1*E*)-1-[1-(3-Hydroxypropyl)-2-pyrrolidinylidene]-2-propanone (**8a**)

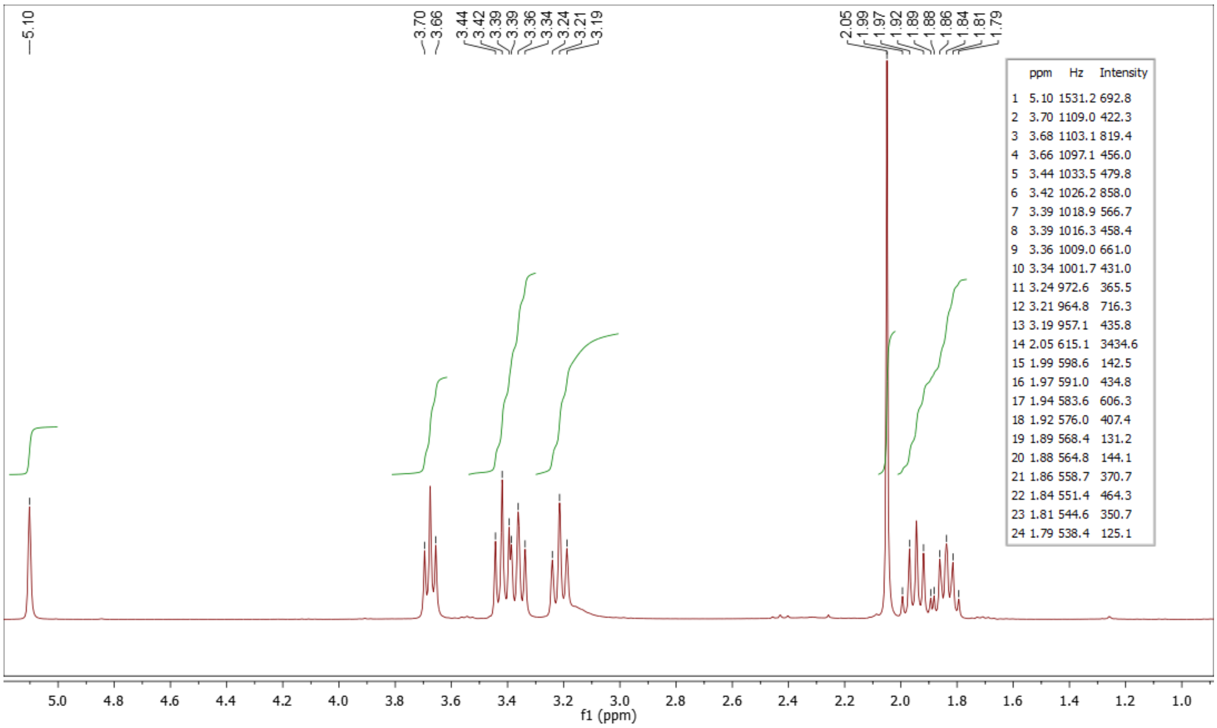

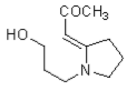

(1*E*)-1-[1-(3-Hydroxypropyl)-2-pyrrolidinylidene]-2-propanone (**8a**)

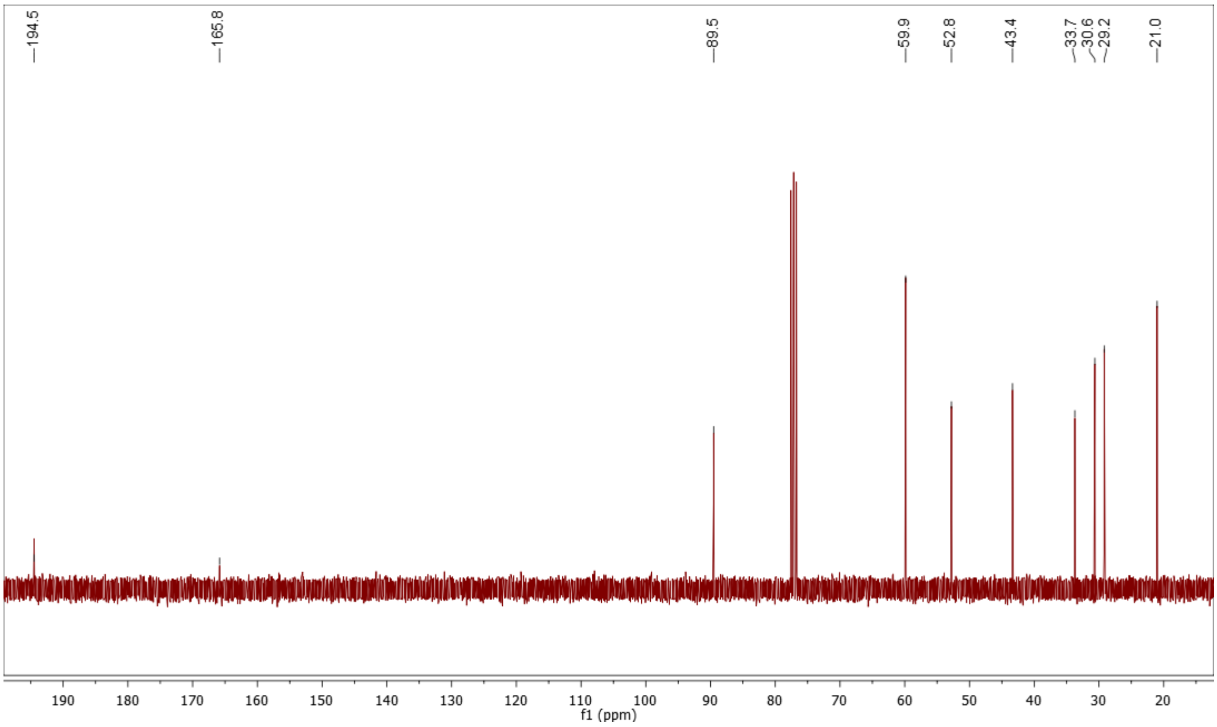

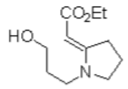

Ethyl (2*E*)-[1-(3-hydroxypropyl)-2-pyrrolidinylidene]ethanoate (**8b**)

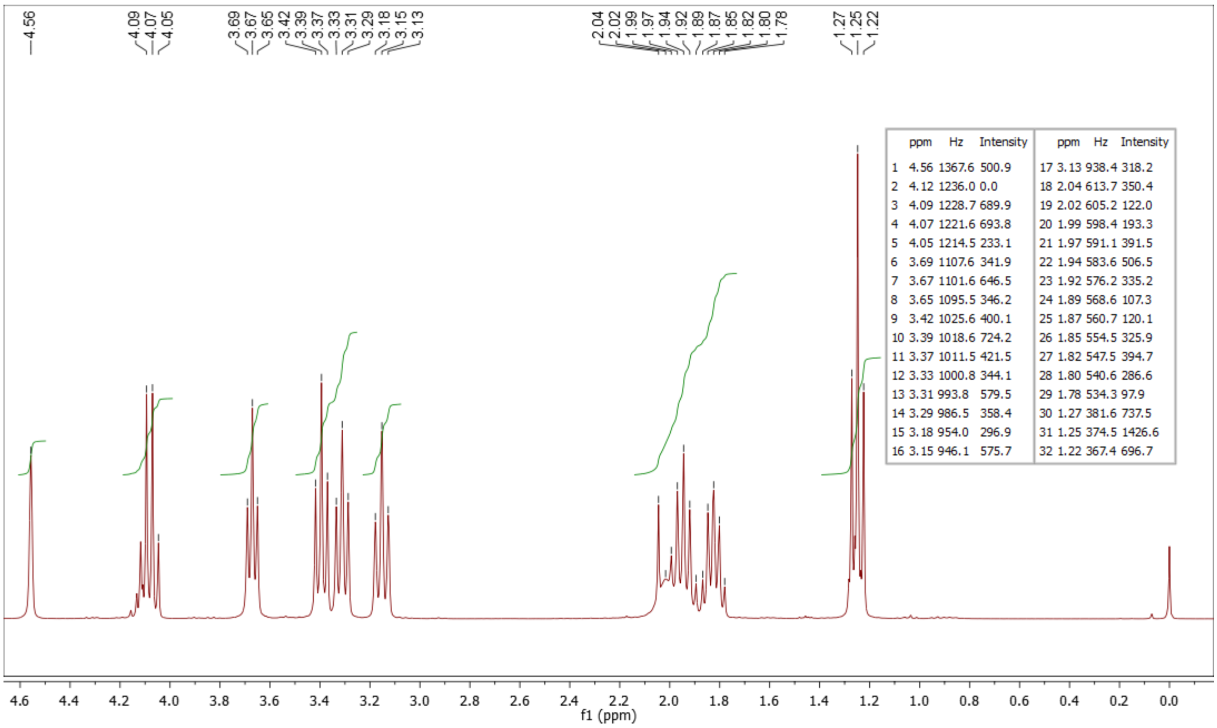

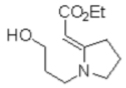

Ethyl (2*E*)-[1-(3-hydroxypropyl)-2-pyrrolidinylidene]ethanoate (**8b**)

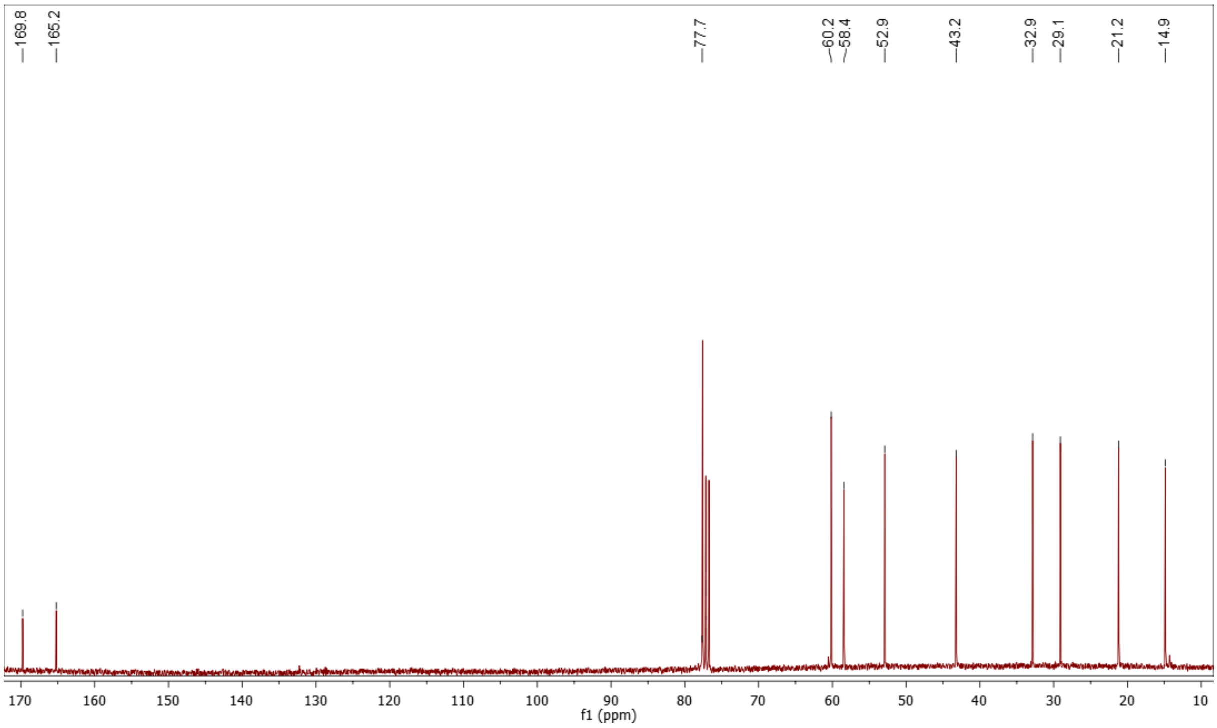

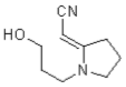

(2*E*)-[1-(3-Hydroxypropyl)-2-pyrrolidinylidene]ethanenitrile (**8c**)

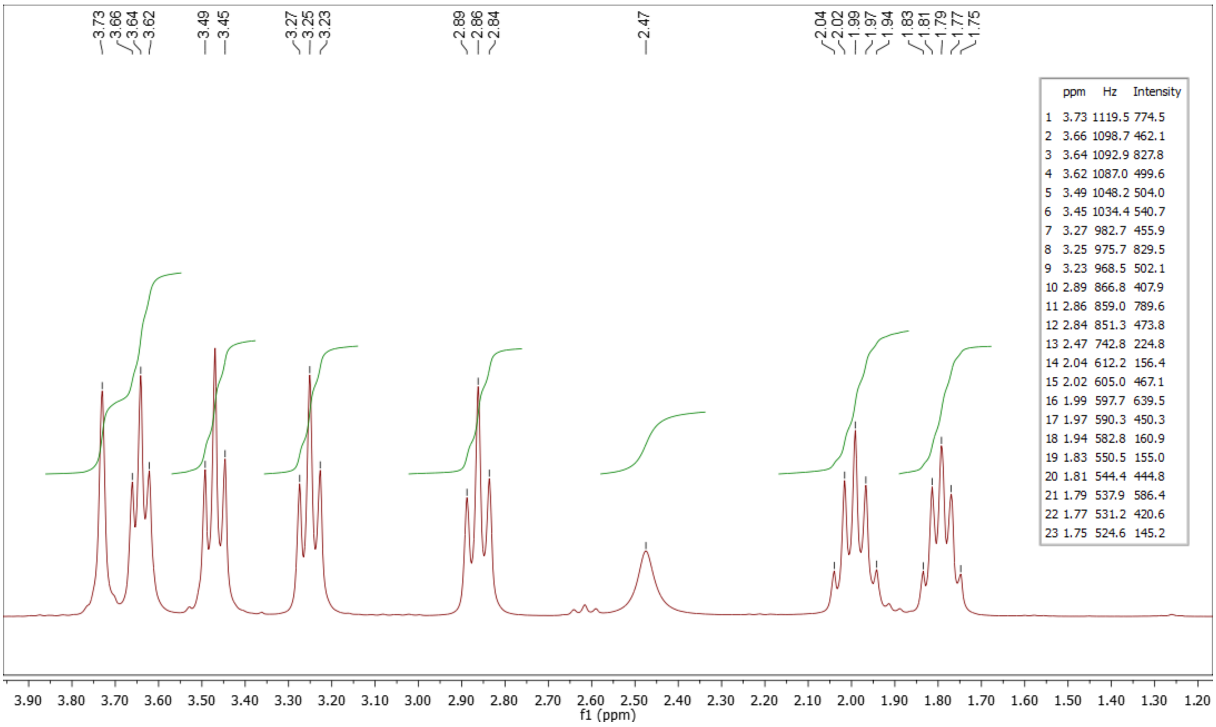

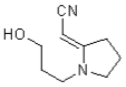

(2*E*)-[1-(3-Hydroxypropyl)-2-pyrrolidinylidene]ethanenitrile (**8c**)

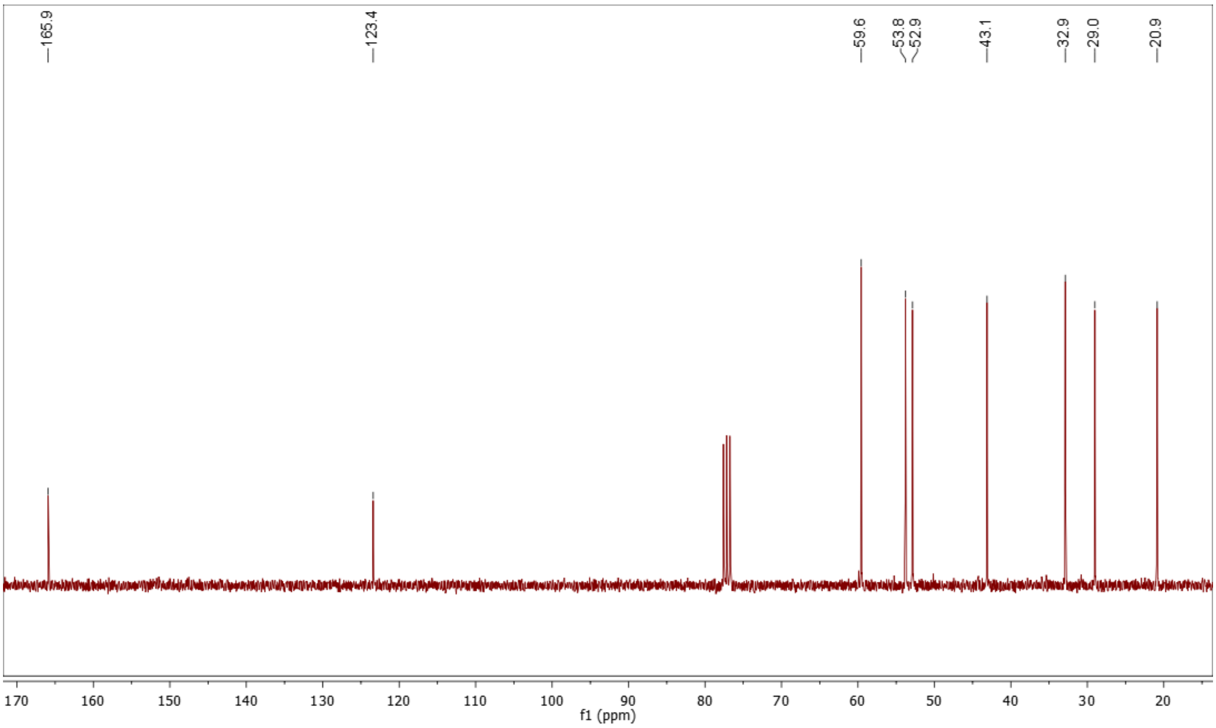

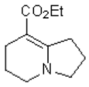

Ethyl 1,2,3,5,6,7-hexahydroindolizine-8-carboxylate (**9b**)

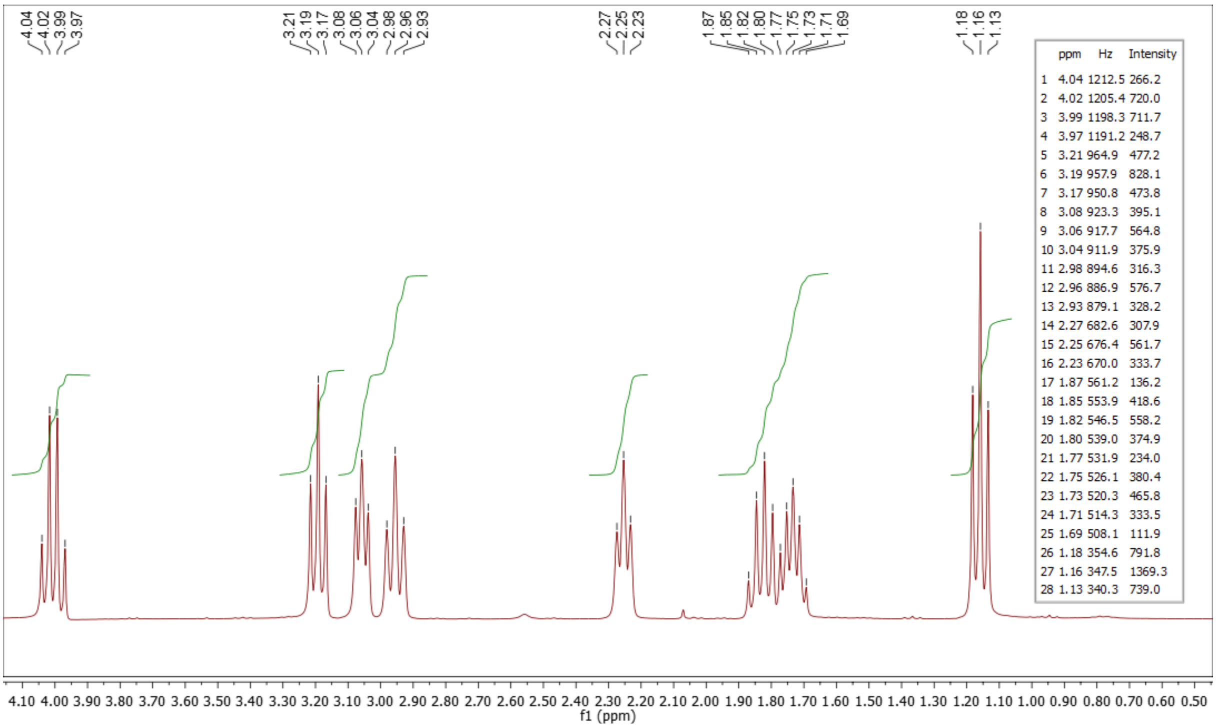

	ppm	Hz	Intensity
1	4.04	1212.5	266.2
2	4.02	1205.4	720.0
3	3.99	1198.3	711.7
4	3.97	1191.2	248.7
5	3.21	964.9	477.2
6	3.19	957.9	828.1
7	3.17	950.8	473.8
8	3.08	923.3	395.1
9	3.06	917.7	564.8
10	3.04	911.9	375.9
11	2.98	894.6	316.3
12	2.96	886.9	576.7
13	2.93	879.1	328.2
14	2.27	682.6	307.9
15	2.25	676.4	561.7
16	2.23	670.0	333.7
17	1.87	561.2	136.2
18	1.85	553.9	418.6
19	1.82	546.5	558.2
20	1.80	539.0	374.9
21	1.77	531.9	234.0
22	1.75	526.1	380.4
23	1.73	520.3	465.8
24	1.71	514.3	333.5
25	1.69	508.1	111.9
26	1.18	354.6	791.8
27	1.16	347.5	1369.3
28	1.13	340.3	739.0

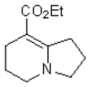

Ethyl 1,2,3,5,6,7-hexahydroindolizine-8-carboxylate (**9b**)

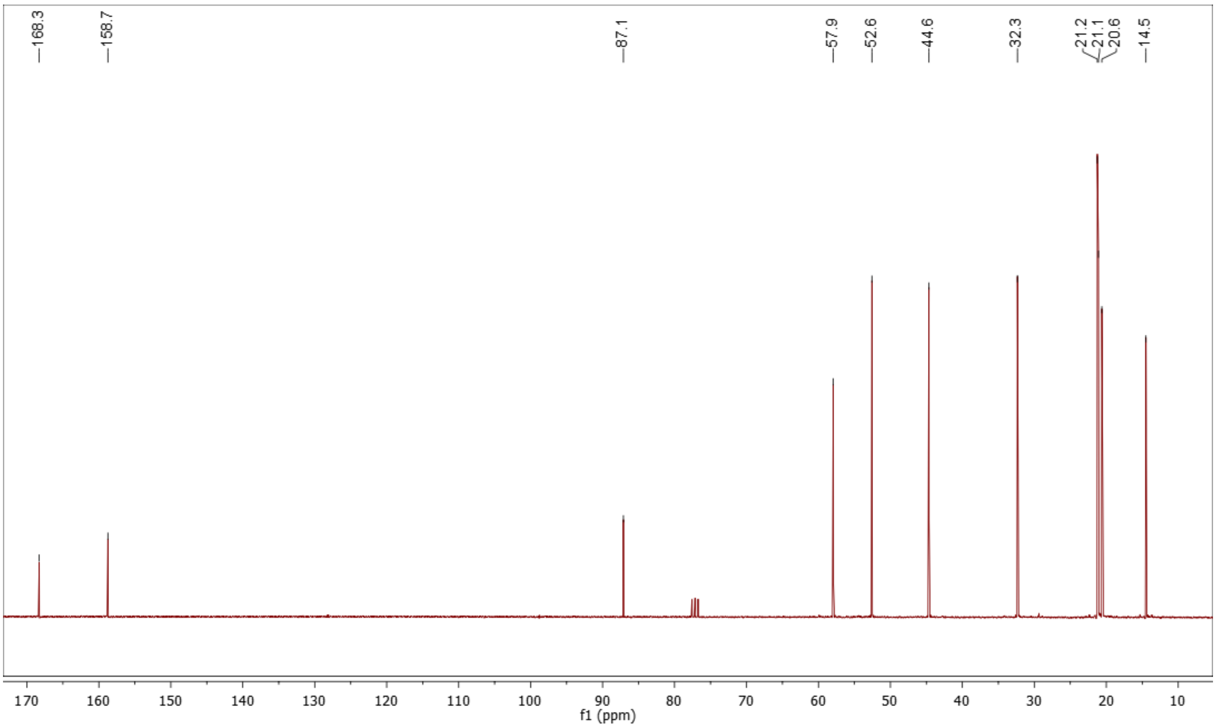

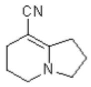

1,2,3,5,6,7-Hexahydroindolizine-8-carbonitrile (**9c**)

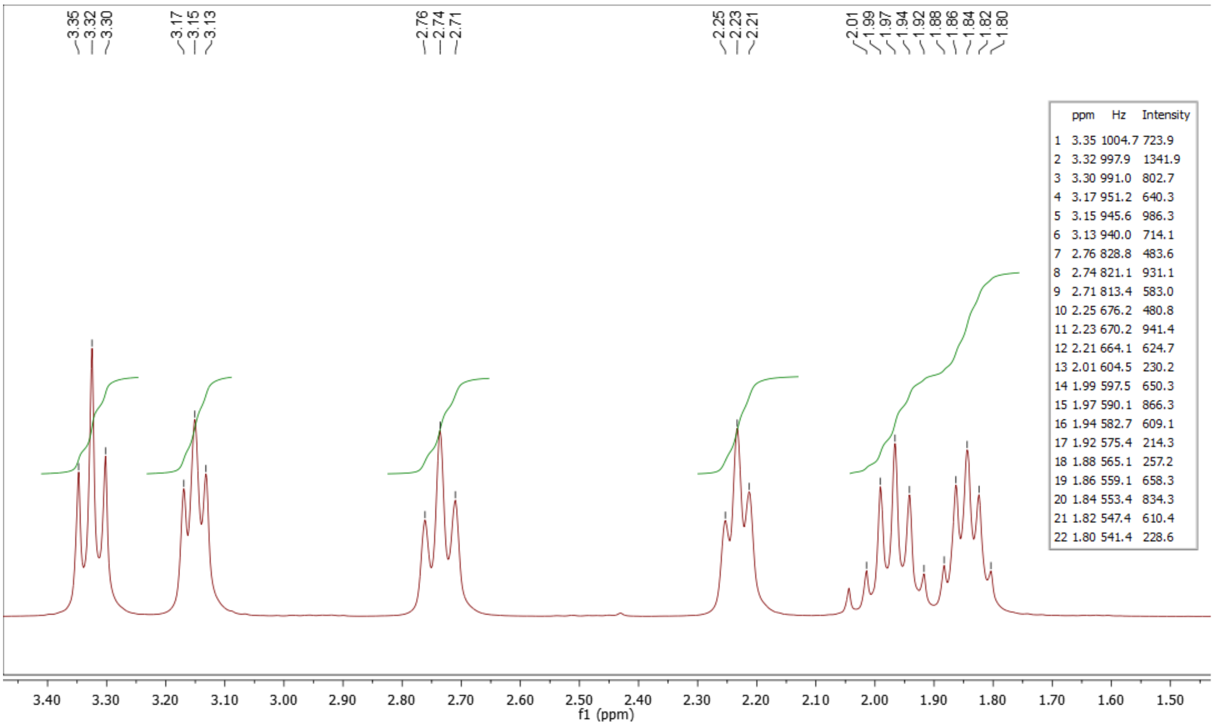

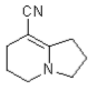

1,2,3,5,6,7-Hexahydroindolizine-8-carbonitrile (**9c**)

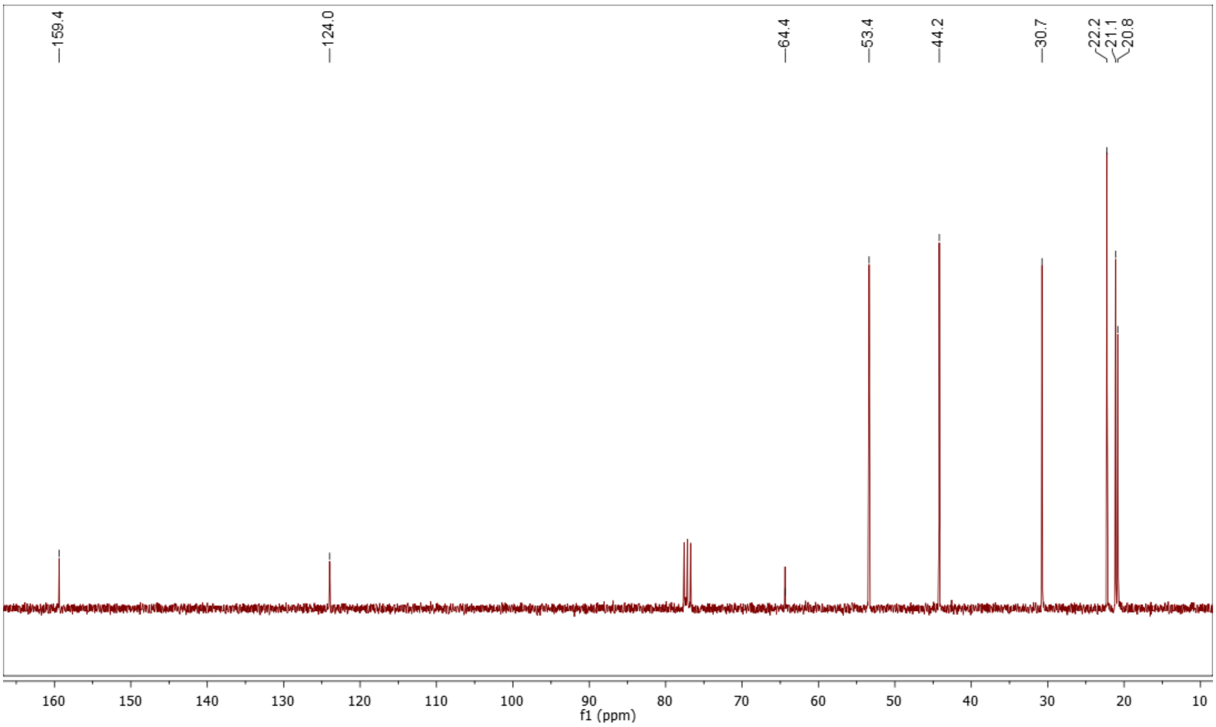

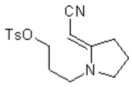

3-[(2*E*)-2-(Cyanomethylene)pyrrolidinyl]propyl 4-methylbenzenesulfonate (**10c**)

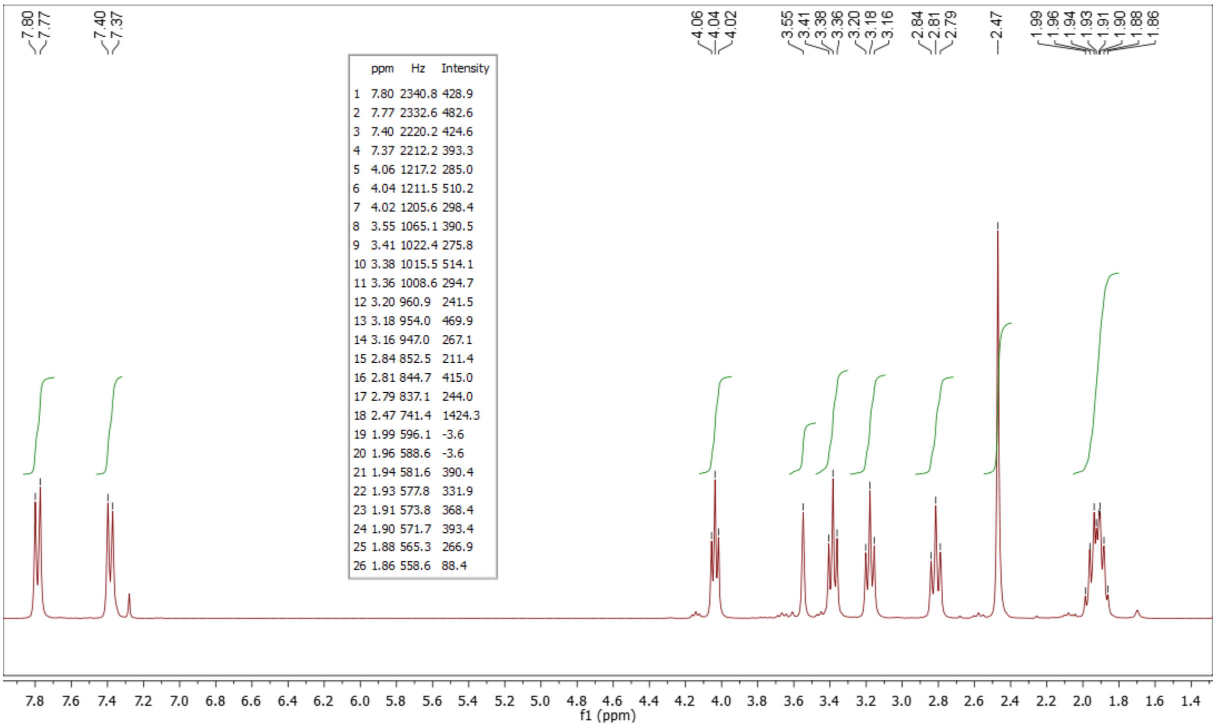

	ppm	Hz	Intensity
1	7.80	2340.8	428.9
2	7.77	2332.6	482.6
3	7.40	2220.2	424.6
4	7.37	2212.2	393.3
5	4.06	1217.2	285.0
6	4.04	1211.5	510.2
7	4.02	1205.6	298.4
8	3.55	1065.1	390.5
9	3.41	1022.4	275.8
10	3.38	1015.5	514.1
11	3.36	1008.6	294.7
12	3.20	960.9	241.5
13	3.18	954.0	469.9
14	3.16	947.0	267.1
15	2.84	852.5	211.4
16	2.81	844.7	415.0
17	2.79	837.1	244.0
18	2.47	741.4	1424.3
19	1.99	596.1	-3.6
20	1.96	588.6	-3.6
21	1.94	581.6	390.4
22	1.93	577.8	331.9
23	1.91	573.8	368.4
24	1.90	571.7	393.4
25	1.88	565.3	266.9
26	1.86	558.6	88.4

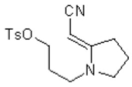

3-[(2*E*)-2-(Cyanomethylene)pyrrolidinyl]propyl 4-methylbenzenesulfonate (**10c**)

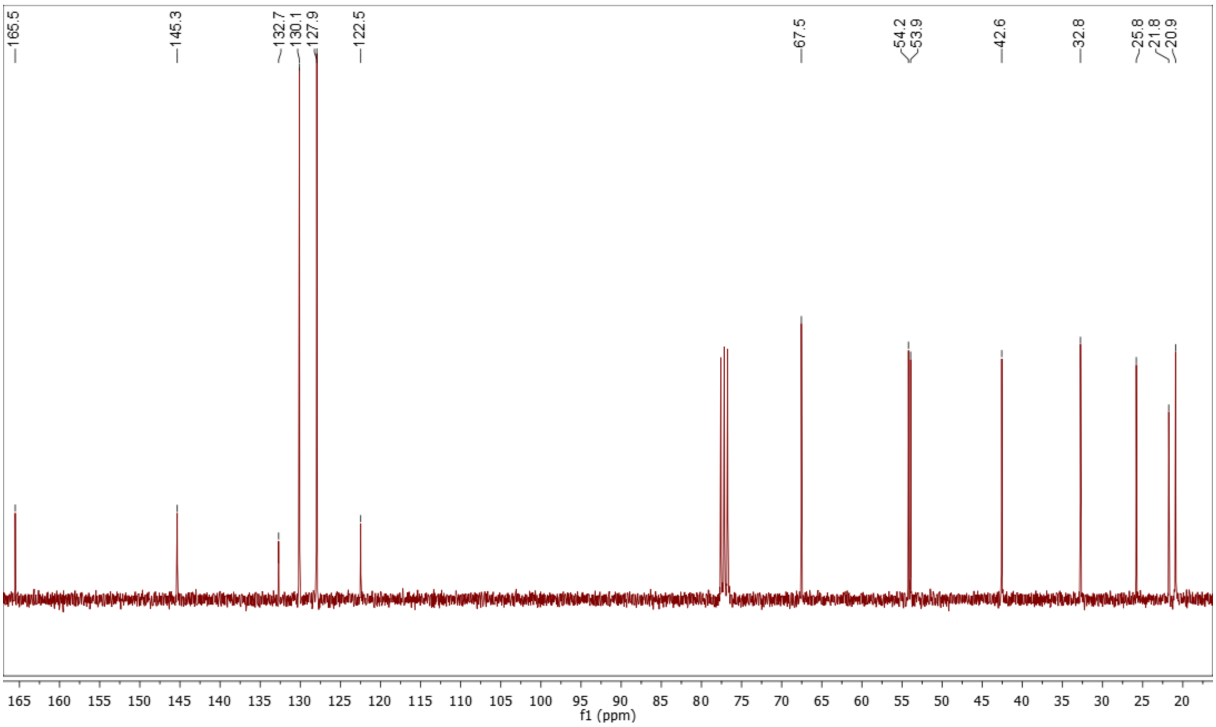

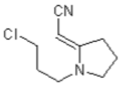

(2*E*)-[1-(3-Chloropropyl)-2-pyrrolidinylidene]ethanenitrile (**11c**)

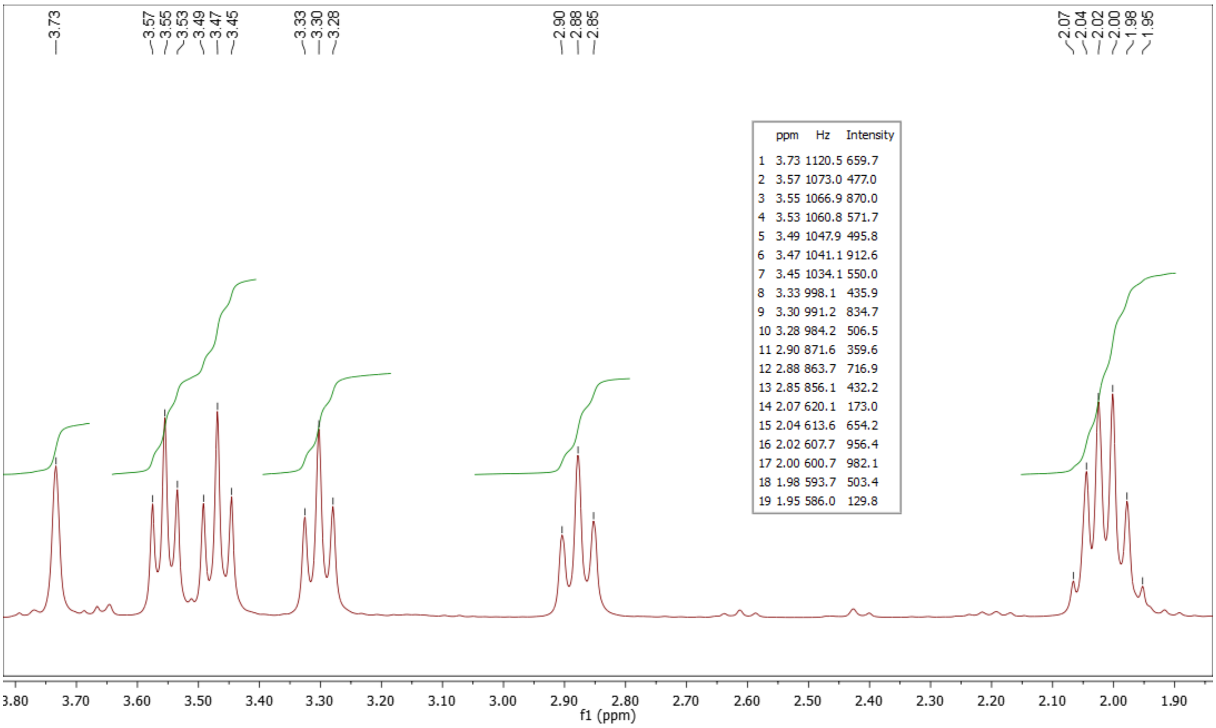

	ppm	Hz	Intensity
1	3.73	1120.5	659.7
2	3.57	1073.0	477.0
3	3.55	1066.9	870.0
4	3.53	1060.8	571.7
5	3.49	1047.9	495.8
6	3.47	1041.1	912.6
7	3.45	1034.1	550.0
8	3.33	998.1	435.9
9	3.30	991.2	834.7
10	3.28	984.2	506.5
11	2.90	871.6	359.6
12	2.88	863.7	716.9
13	2.85	856.1	432.2
14	2.07	620.1	173.0
15	2.04	613.6	654.2
16	2.02	607.7	956.4
17	2.00	600.7	982.1
18	1.98	593.7	503.4
19	1.95	586.0	129.8

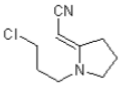

(2*E*)-[1-(3-Chloropropyl)-2-pyrrolidinylidene]ethanenitrile (**11c**)

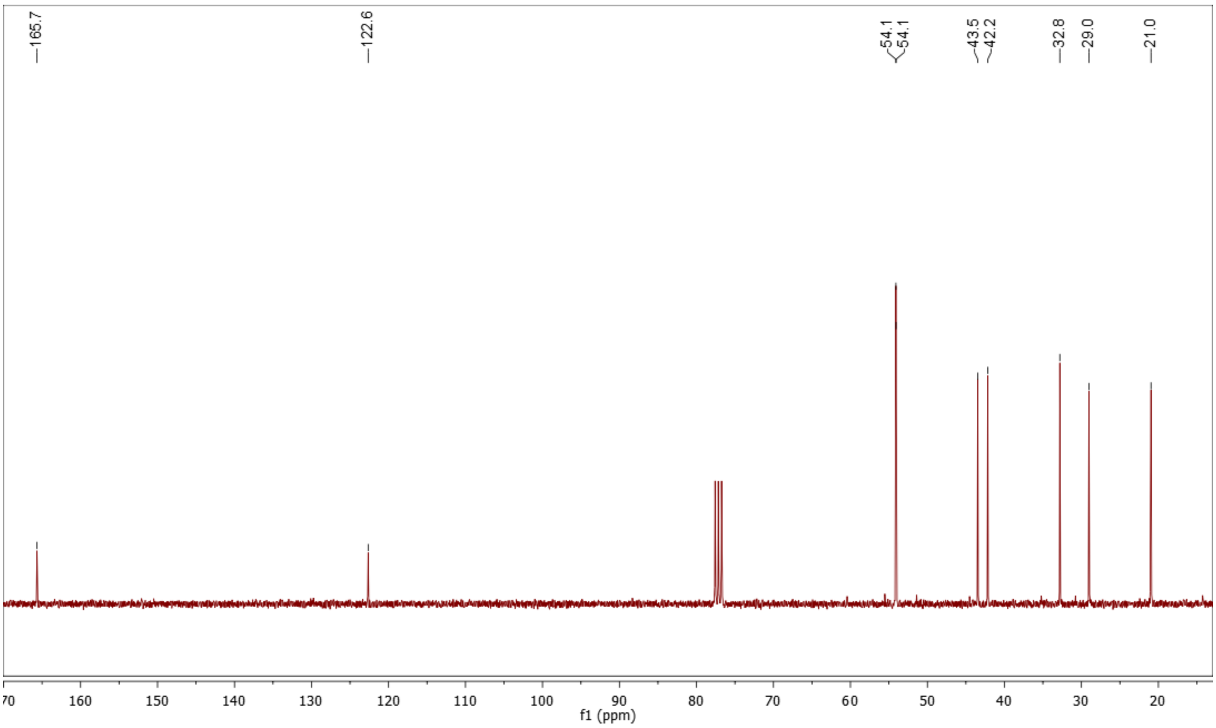

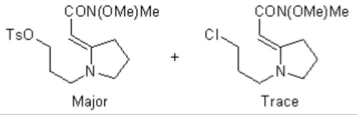

3-((2*E*)-2-{2-[Methoxy(methyl)amino]-2-oxoethylidene}pyrrolidinyl)propyl 4-methylbenzenesulfonate (**10d**) and (2*E*)-2-[1-(3-chloropropyl)-2-pyrrolidinylidene]-*N*-methoxy-*N*-methylethanamide (**11d**)

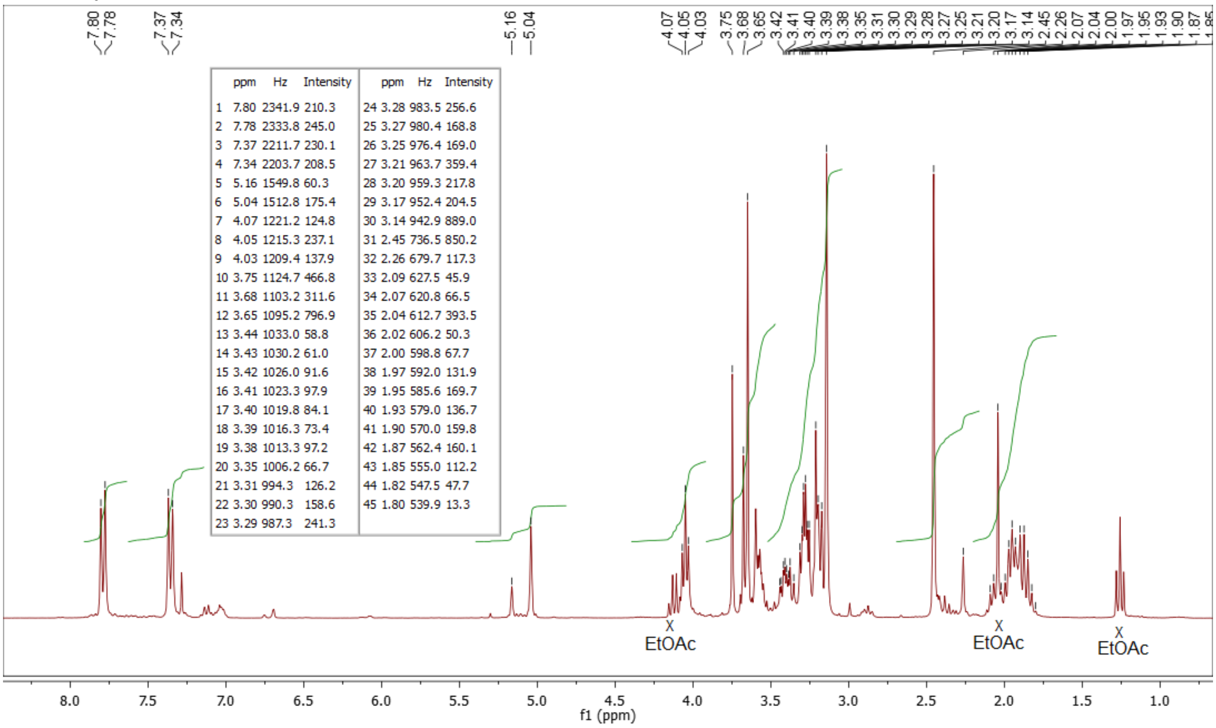

	ppm	Hz	Intensity		ppm	Hz	Intensity
1	7.80	2341.9	210.3	24	3.28	983.5	256.6
2	7.78	2333.8	245.0	25	3.27	980.4	168.8
3	7.37	2211.7	230.1	26	3.25	976.4	169.0
4	7.34	2203.7	208.5	27	3.21	963.7	359.4
5	5.16	1549.8	60.3	28	3.20	959.3	217.8
6	5.04	1512.8	175.4	29	3.17	952.4	204.5
7	4.07	1221.2	124.8	30	3.14	942.9	889.0
8	4.05	1215.3	237.1	31	2.45	736.5	850.2
9	4.03	1209.4	137.9	32	2.26	679.7	117.3
10	3.75	1124.7	466.8	33	2.09	627.5	45.9
11	3.68	1103.2	311.6	34	2.07	620.8	66.5
12	3.65	1095.2	796.9	35	2.04	612.7	393.5
13	3.44	1033.0	58.8	36	2.02	606.2	50.3
14	3.43	1030.2	61.0	37	2.00	598.8	67.7
15	3.42	1026.0	91.6	38	1.97	592.0	131.9
16	3.41	1023.3	97.9	39	1.95	585.6	169.7
17	3.40	1019.8	84.1	40	1.93	579.0	136.7
18	3.39	1016.3	73.4	41	1.90	570.0	159.8
19	3.38	1013.3	97.2	42	1.87	562.4	160.1
20	3.35	1006.2	66.7	43	1.85	555.0	112.2
21	3.31	994.3	126.2	44	1.82	547.5	47.7
22	3.30	990.3	158.6	45	1.80	539.9	13.3
23	3.29	987.3	241.3				

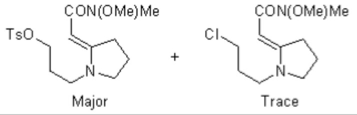

3-((2*E*)-2-{2-[Methoxy(methyl)amino]-2-oxoethylidene}pyrrolidinyl)propyl 4-methylbenzenesulfonate (**10d**) and (2*E*)-2-[1-(3-chloropropyl)-2-pyrrolidinylidene]-*N*-methoxy-*N*-methylethanamide (**11d**)

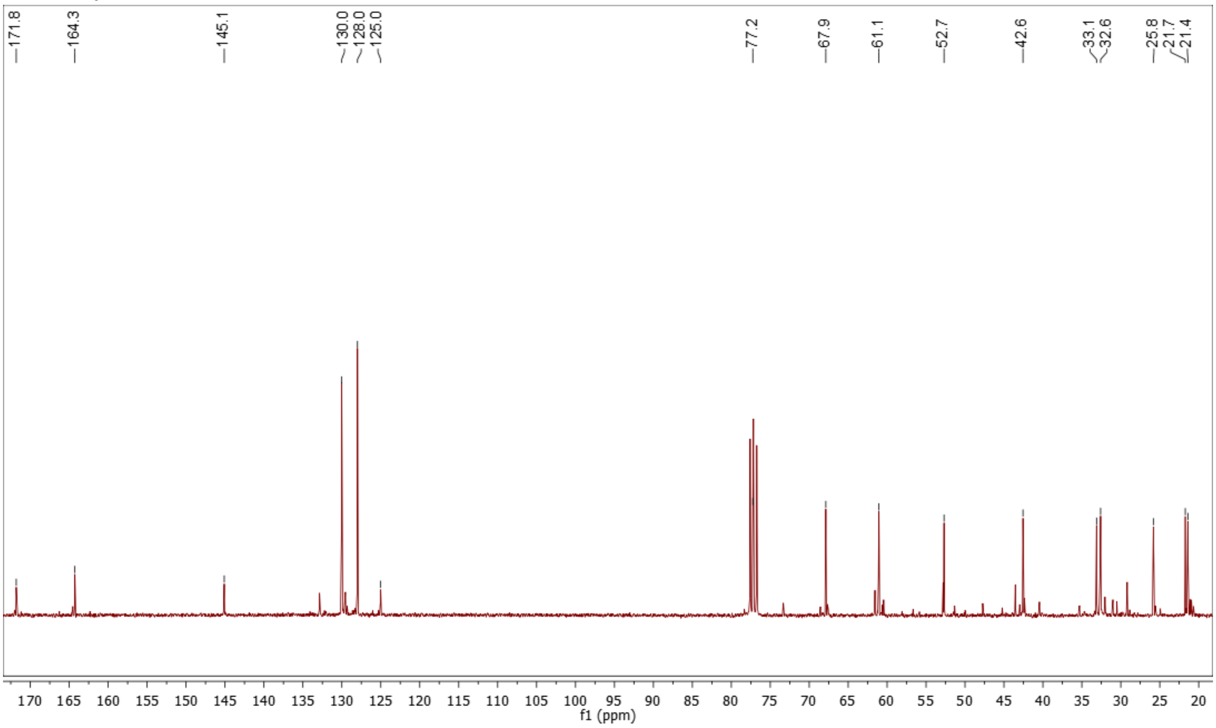

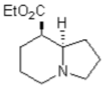

Ethyl (8*R**,8a*R**)-octahydroindolizine-8-carboxylate (**12b'**)

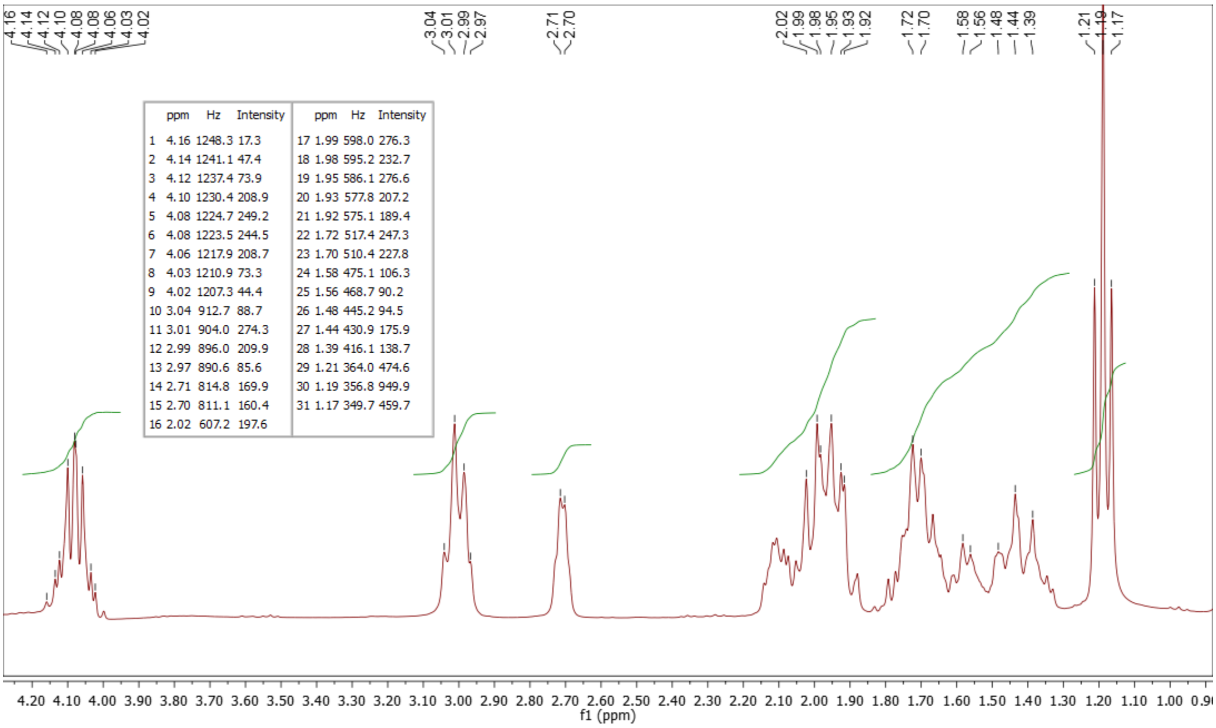

	ppm	Hz	Intensity		ppm	Hz	Intensity
1	4.16	1248.3	17.3	17	1.99	598.0	276.3
2	4.14	1241.1	47.4	18	1.98	595.2	232.7
3	4.12	1237.4	73.9	19	1.95	586.1	276.6
4	4.10	1230.4	208.9	20	1.93	577.8	207.2
5	4.08	1224.7	249.2	21	1.92	575.1	189.4
6	4.08	1223.5	244.5	22	1.72	517.4	247.3
7	4.06	1217.9	208.7	23	1.70	510.4	227.8
8	4.03	1210.9	73.3	24	1.58	475.1	106.3
9	4.02	1207.3	44.4	25	1.56	468.7	90.2
10	3.04	912.7	88.7	26	1.48	445.2	94.5
11	3.01	904.0	274.3	27	1.44	430.9	175.9
12	2.99	896.0	209.9	28	1.39	416.1	138.7
13	2.97	890.6	85.6	29	1.21	364.0	474.6
14	2.71	814.8	169.9	30	1.19	356.8	949.9
15	2.70	811.1	160.4	31	1.17	349.7	459.7
16	2.02	607.2	197.6				

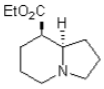

Ethyl (8*R**,8a*R**)-octahydroindolizine-8-carboxylate (**12b'**)

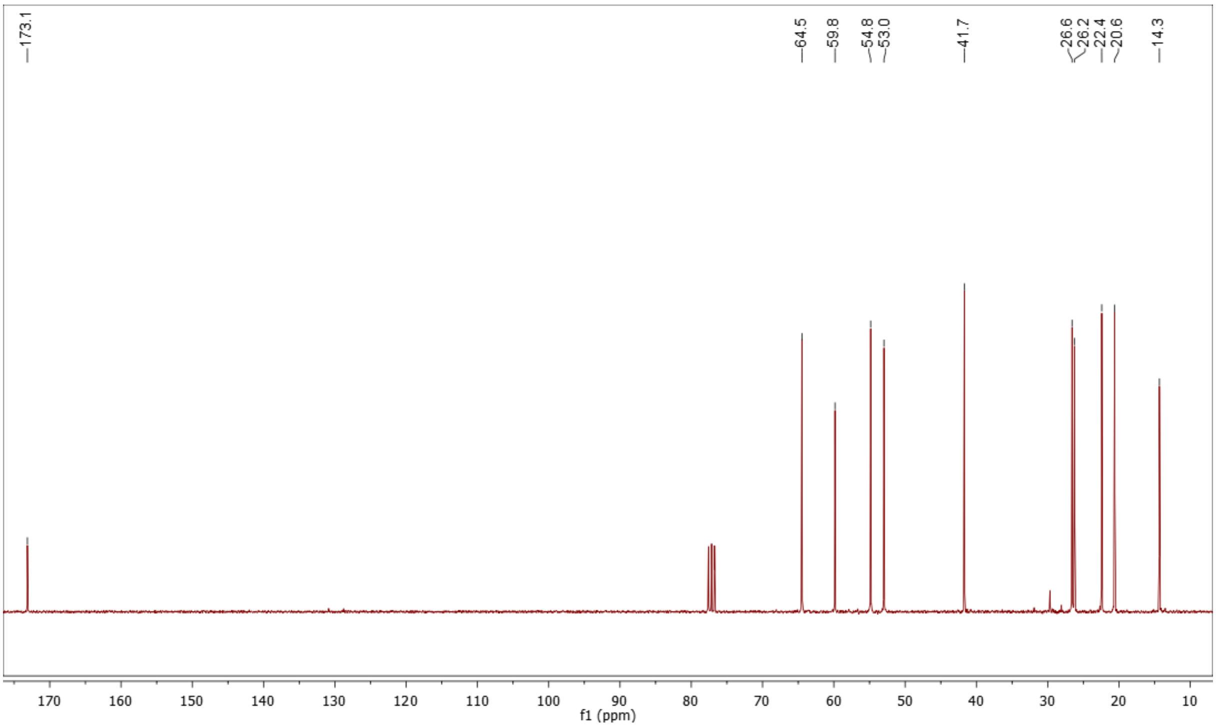

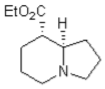

Ethyl (8*R**,8a*S**)-octahydroindolizine-8-carboxylate (**12b''**)

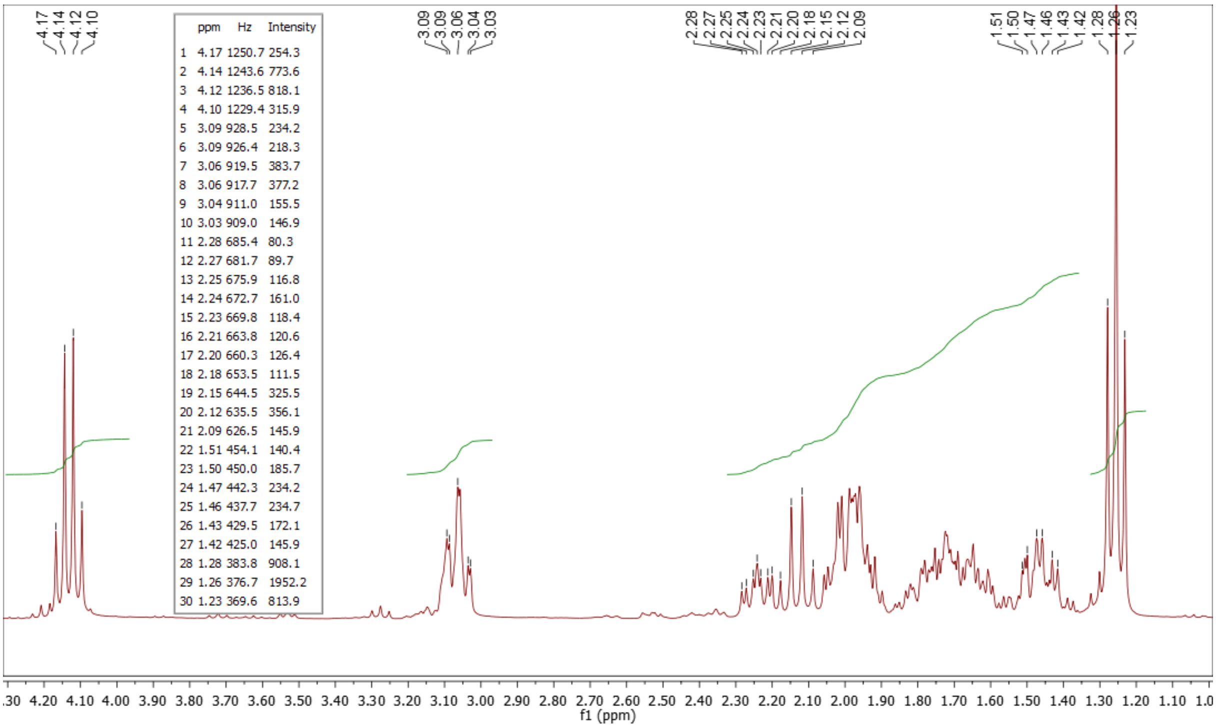

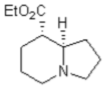

Ethyl (8*R**,8a*S**)-octahydroindolizine-8-carboxylate (**12b''**)

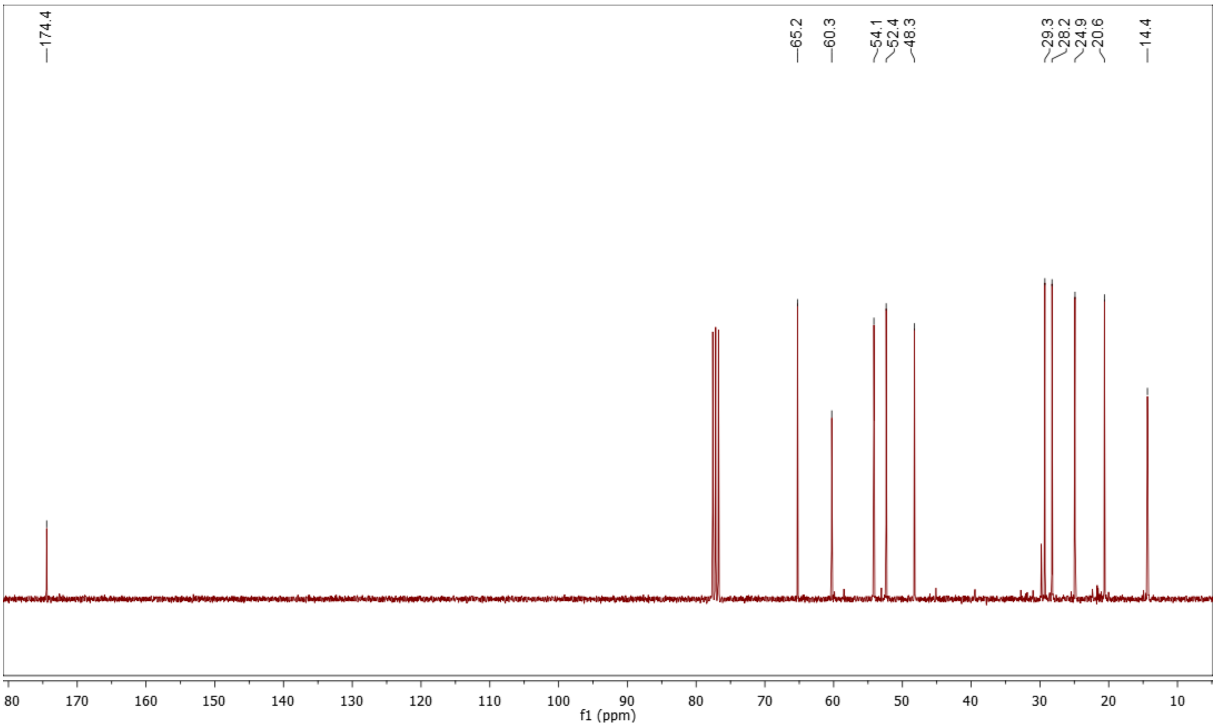

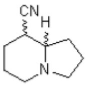

Octahydroindolizine-8-carbonitrile diastereomers (**12c**)

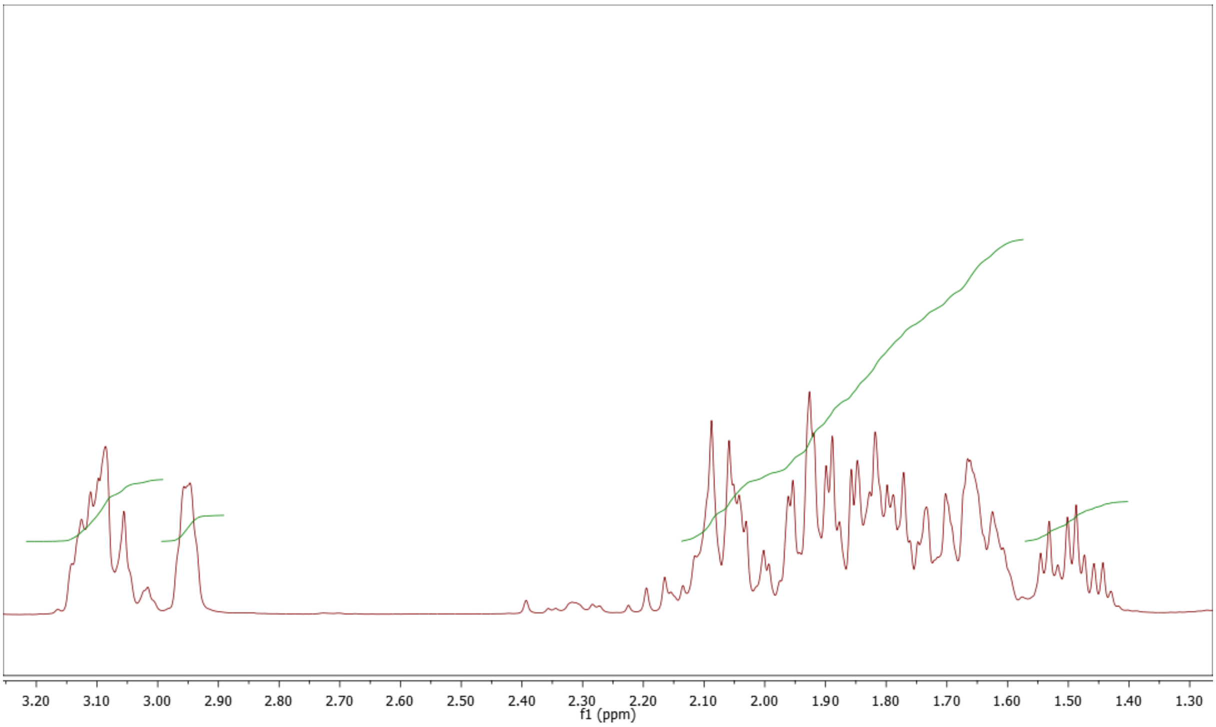

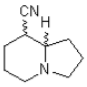

Octahydroindolizine-8-carbonitrile diastereomers (**12c**)

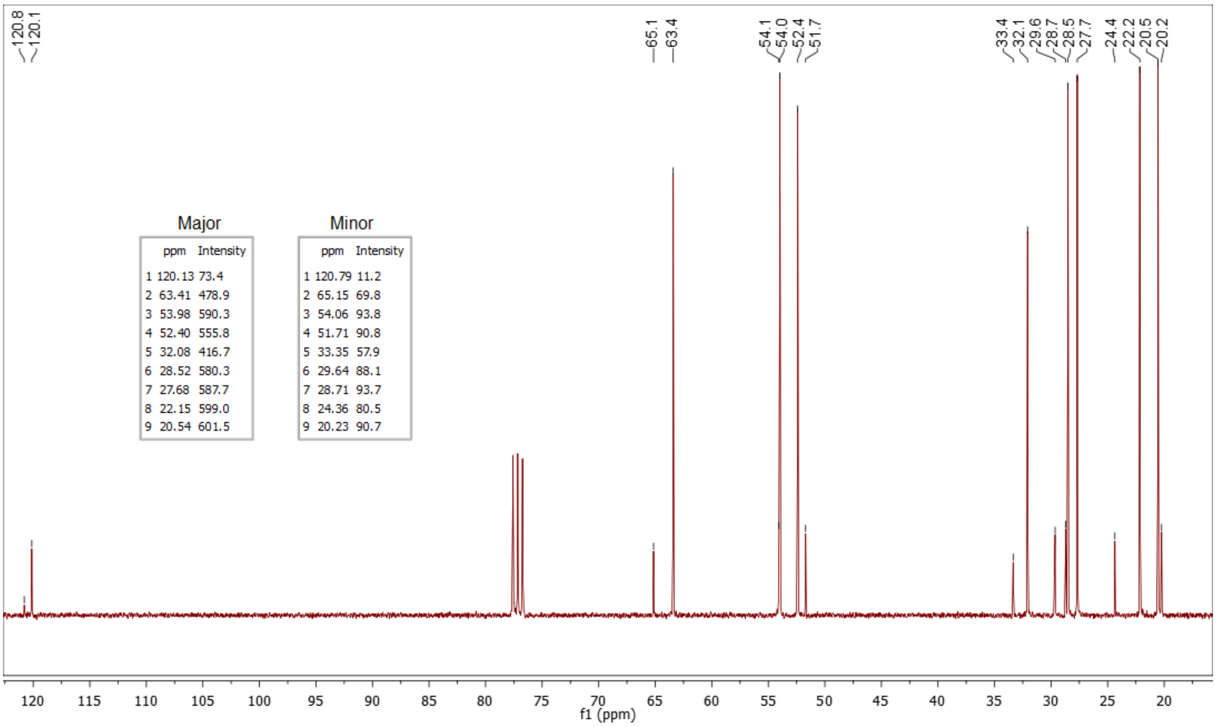

Major

	ppm	Intensity
1	120.13	73.4
2	63.41	478.9
3	53.98	590.3
4	52.40	555.8
5	32.08	416.7
6	28.52	580.3
7	27.68	587.7
8	22.15	599.0
9	20.54	601.5

Minor

	ppm	Intensity
1	120.79	11.2
2	65.15	69.8
3	54.06	93.8
4	51.71	90.8
5	33.35	57.9
6	29.64	88.1
7	28.71	93.7
8	24.36	80.5
9	20.23	90.7

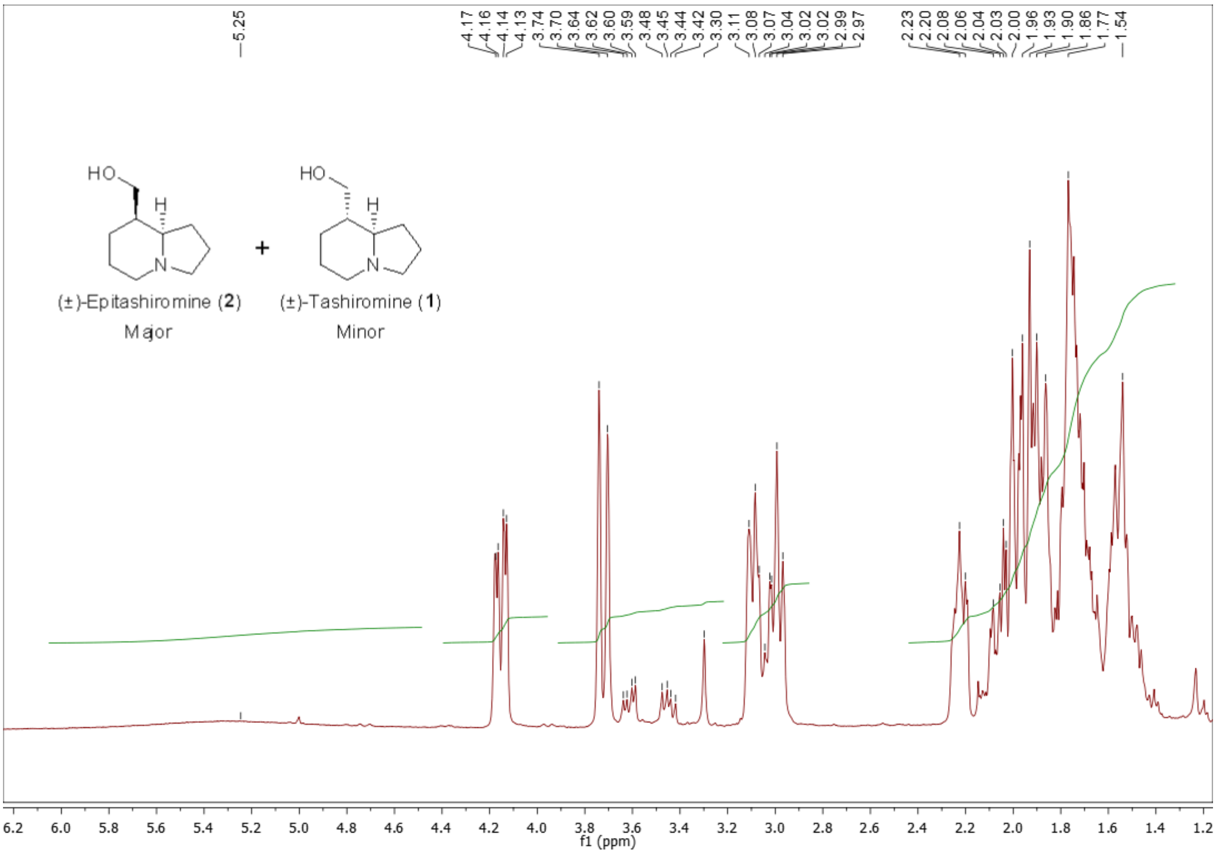
5.25
4.17 4.16 4.14 4.13 3.74 3.70 3.64 3.62 3.60 3.59 3.48 3.45 3.44 3.42 3.30 3.11 3.08 3.07 3.04 3.02 3.02 2.99 2.97
2.23 2.20 2.08 2.06 2.04 2.03 2.00 1.96 1.93 1.90 1.86 1.77 1.54
HO
H
N
+
(±)-Epitashiromine (2)
Major
(±)-Tashiromine (1)
Minor
6.2 6.0 5.8 5.6 5.4 5.2 5.0 4.8 4.6 4.4 4.2 4.0 3.8 3.6 3.4 3.2 3.0 2.8 2.6 2.4 2.2 2.0 1.8 1.6 1.4 1.2
f1 (ppm)

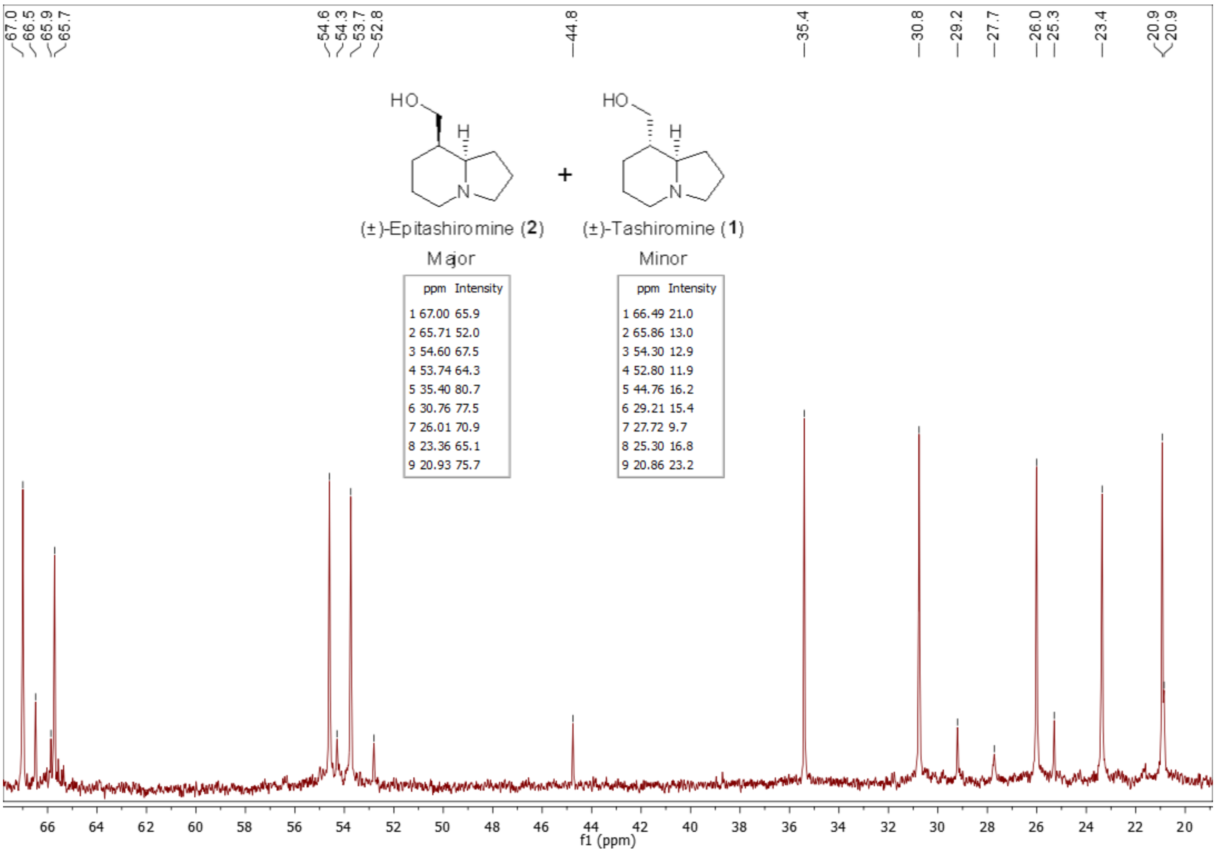
67.0 66.5 65.9 65.7
54.6 54.3 53.7 52.8
44.8
35.4
30.8 29.2 27.7 26.0 25.3 23.4 20.9 20.9
HO
H
N
+
(±)-Epitashiromine (2)
Major
ppm Intensity
1 67.00 65.9
2 65.71 52.0
3 54.60 67.5
4 53.74 64.3
5 35.40 80.7
6 30.76 77.5
7 26.01 70.9
8 23.36 65.1
9 20.93 75.7
(±)-Tashiromine (1)
Minor
ppm Intensity
1 66.49 21.0
2 65.86 13.0
3 54.30 12.9
4 52.80 11.9
5 44.76 16.2
6 29.21 15.4
7 27.72 9.7
8 25.30 16.8
9 20.86 23.2
66 64 62 60 58 56 54 52 50 48 46 44 42 40 38 36 34 32 30 28 26 24 22 20
f1 (ppm)

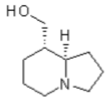

(±)-Tashiromine (**1**)

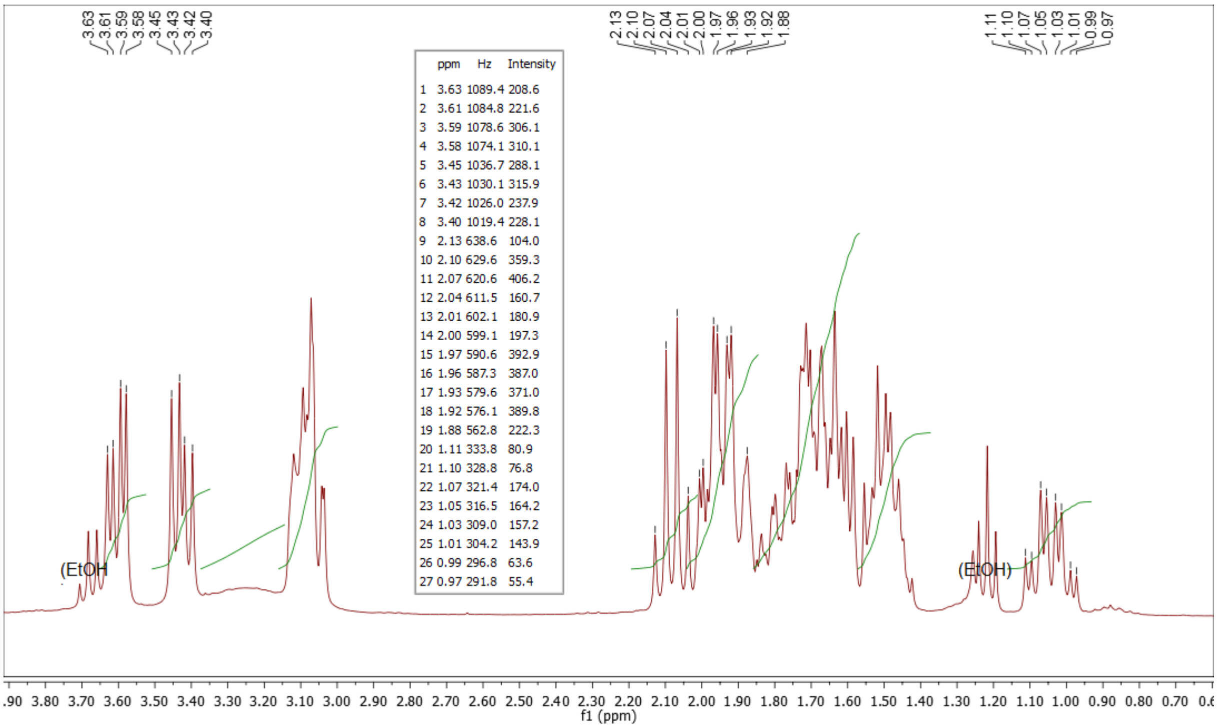

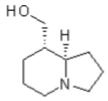

(±)-Tashiromine (**1**)

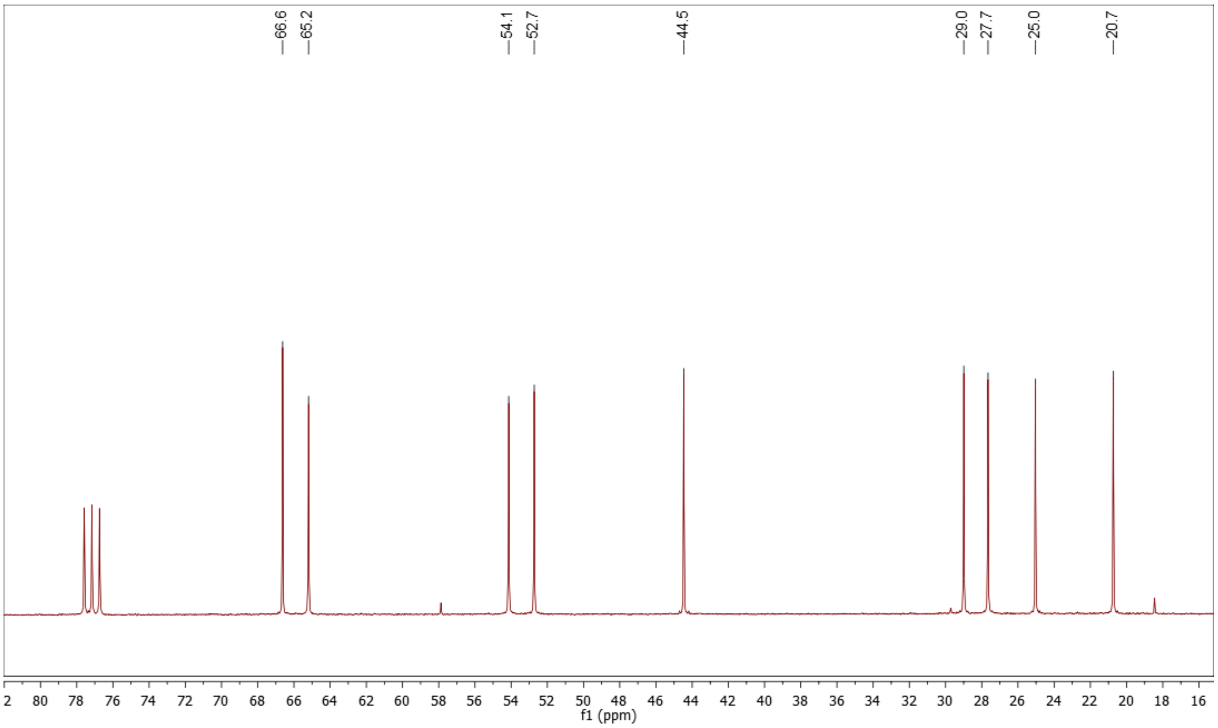

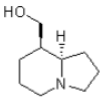

(±)-Epitashiromine (**2**)

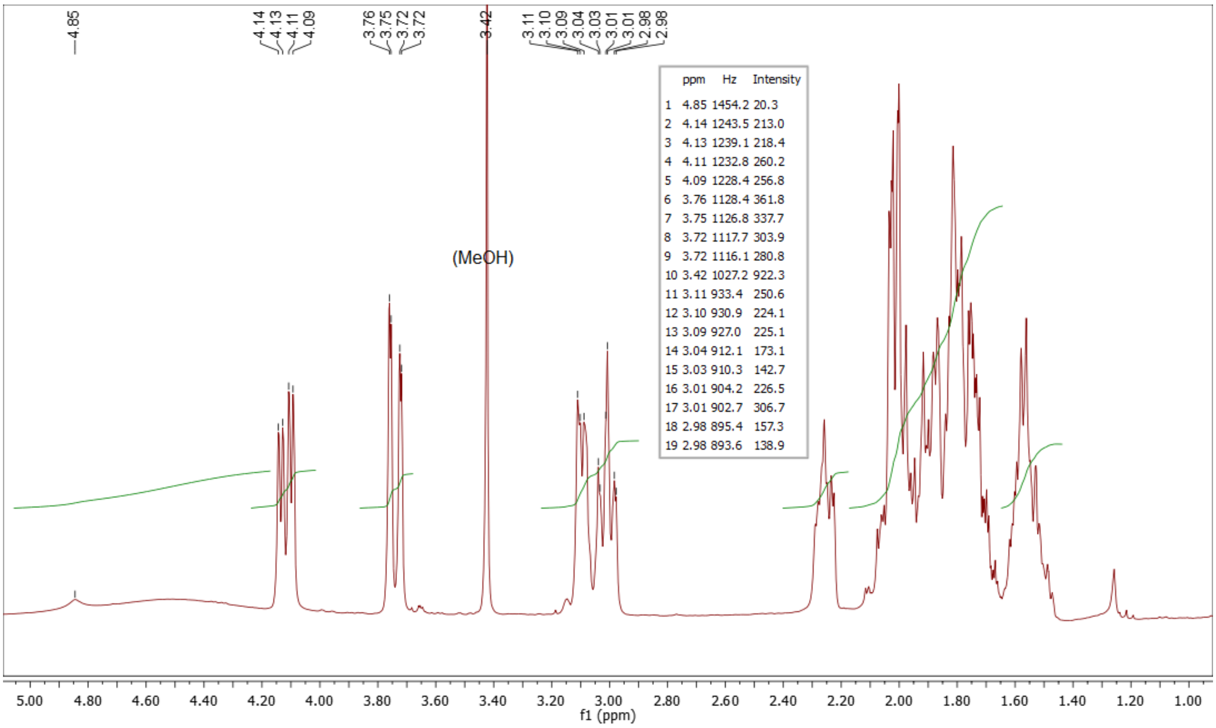

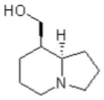

(±)-Epitashiromine (**2**)

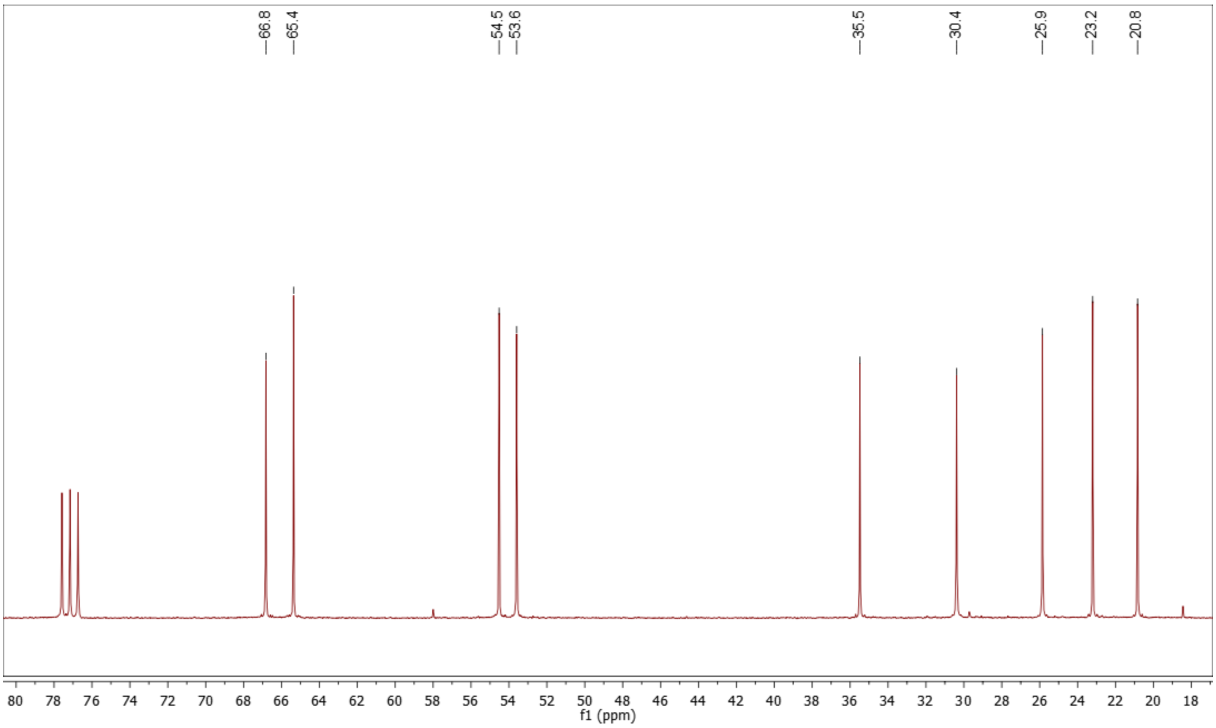

Supplement: File 1 — Experimental procedures and copies of NMR spectra. [file Beilstein_J_Org_Chem-12-2609-s001.pdf]
